# Re-evaluation of the causes of variation among mouse aggregation chimaeras

**DOI:** 10.1242/bio.042804

**Published:** 2019-05-15

**Authors:** John D. West, Pin-Chi Tang, Clare A. Everett, Gillian E. MacKay, Jean H. Flockhart, Margaret A. Keighren

**Affiliations:** Genes and Development Group, Centre for Integrative Physiology, Clinical Sciences, University of Edinburgh Medical School, Hugh Robson Building, George Square, Edinburgh EH8 9XD, UK

**Keywords:** Chimaera, Chimera, Developmental lineages, Cell allocation, Mouse, Variation in composition

## Abstract

The composition of adult mouse aggregation chimaeras is much more variable than X-inactivation mosaics. An early theoretical model proposed that almost all the extra variation in chimaeras arises, before X-inactivation occurs, by spatially constrained, geometrical allocation of inner cell mass (ICM) cells to the epiblast and primitive endoderm (PrE). However, this is inconsistent with more recent embryological evidence. Analysis of published results for chimaeric blastocysts and mid-gestation chimaeras suggested that some variation exists among chimaeric morulae and more variation arises both when morula cells are allocated to the ICM versus the trophectoderm (TE) and when ICM cells are allocated to the epiblast versus the PrE. Computer simulation results were also consistent with the conclusion that stochastic allocation of cells to blastocyst lineages in two steps, without the type of geometrical sampling that was originally proposed, could cause a wide variation in chimaeric epiblast composition. Later allocation events will cause additional variation among both chimaeras and X-inactivation mosaics. We also suggest that previously published U-shaped frequency distributions for chimaeric placenta composition might be explained by how TE cells are allocated to the polar TE and/or the subsequent movement of cells from polar TE to mural TE.

## Abstract (precis)

**Summary:** Analysis of published results suggests that two stochastic allocation steps in chimaeric mouse blastocysts, without the geometrical sampling mechanism proposed previously, could explain much of the variation among aggregation chimaeras.

## INTRODUCTION

Mouse aggregation chimaeras and mouse X-inactivation mosaics both comprise two distinct cell populations. Mouse aggregation chimaeras are typically produced by aggregating two genetically distinct eight-cell-stage mouse embryos (Tarkowski, 1961) and so comprise two genetically distinct cell populations from the time of aggregation. Mouse X-inactivation mosaics are XX females that are heterozygous for one or more X-linked marker genes. After random inactivation of one of the two X chromosomes in the epiblast lineage, some cells express only the maternally-derived X-chromosome and others express only the paternally-derived X-chromosome (Lyon, 1961). Thus, these female mice are functional mosaics for expression of the heterozygous X-linked marker genes from the time that X-chromosome inactivation occurs in the epiblast. The composition of mouse aggregation chimaeras varies much more widely than that of X-inactivation mosaics for both adults (Mullen and Whitten, 1971; Falconer and Avery, 1978) and foetuses (West et al., 1984). This is usually attributed to sampling events that occur in preimplantation stage chimaeras and so precede random X-chromosome inactivation, which begins in the epiblast lineage soon after implantation at around embryonic day 5.5 (E5.5) (Monk and Harper, 1979; Rastan, 1982; Takagi et al., 1982; Mak et al., 2004; Okamoto et al., 2004; Pasque and Plath, 2015).

Falconer and Avery (1978) reported that coat pigmentation in several series of adult, pigmented↔albino aggregation chimaeras tended to follow a broad frequency distribution, where all percentage values of the coat pigmentation marker were equally likely apart from 0% and 100%, which occurred more frequently. The overall distribution was U-shaped and nearly flat between the extreme values. Falconer and Avery proposed that this variability was a consequence of how cells were allocated to the early developmental lineages. They further argued that the first allocation step, when aggregated cells are allocated to the inner cell mass (ICM) versus the trophectoderm (TE), would cause little variation because cells of the two aggregated embryos remained relatively unmixed (Garner and McLaren, 1974).

**Fig. 1.**
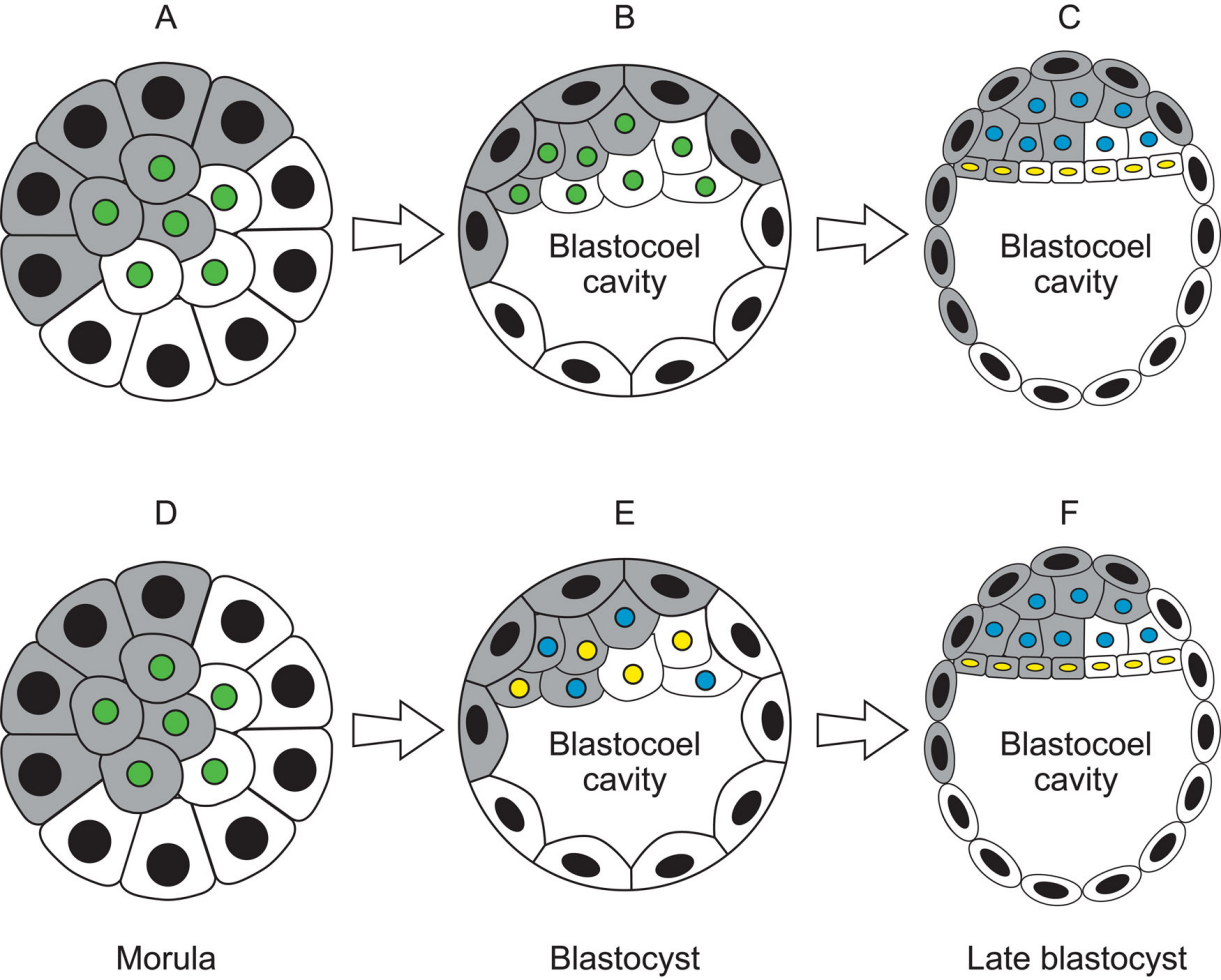
**Two hypotheses predicting how allocation of cells to the three primary lineages in preimplantation stage aggregation chimaeras affects variation in composition of the epiblast lineage.** Diagrams of preimplantation stage aggregation chimaeras, showing allocation of grey and white cell populations to three primary lineages, according to two hypotheses. Cell nuclei are shaded according to their cell type: black, trophectoderm (TE); green, inner cell mass (ICM); blue, epiblast or ICM cell specified to become epiblast; yellow, primitive endoderm (PrE) or ICM cell specified to become PrE. (A–C) Falconer and Avery (1978) proposed that most of the variability among epiblasts in chimaeric blastocysts arises in a single step when ICM cells are allocated to the epiblast or PrE. At the morula stage (A), the aggregated grey and white cells form two hemispheres. It was assumed that, for geometrical reasons, concentric spheres of inner and outer cells would both have approximately equal proportions of grey and white cells. When the morula becomes a blastocyst (B), the inner and outer cells become the ICM and TE respectively. Thus ICM and TE cells also have approximately equal proportions of grey and white cells. At the late blastocyst stage (C) the ICM forms the epiblast and PrE. It was thought that the deeper ICM cells form the epiblast and the ICM cells adjacent to the blastocoel cavity form the PrE. Grey and white ICM cells would remain largely unmixed (Garner and McLaren, 1974) and it was proposed that the proportion of grey epiblast cells would vary according to the angle at which the border between the epiblast and PrE intersects the grey-white boundary. (D–F) An alternative hypothesis proposes that variability among chimaeric epiblasts arises both when morula cells are allocated to the ICM or TE and when ICM cells are allocated to the epiblast or PrE. Thus, it is proposed that grey cells will often contribute unequally to the inner and outer cells at the morula stage (D), so the composition of the ICM and TE may differ. There is now evidence that ICM cells gradually become specified as presumptive epiblast or PrE, at the early to mid-blastocyst stage, and are initially mixed together in a ‘salt and pepper’ distribution (E). Cell specification is completed at the late blastocyst stage (F) and presumptive epiblast and PrE cells move to their final positions and differentiate (see the Introduction for references). Thus, allocation of cells to the ICM or TE and stochastic specification of ICM cells as epiblast or PrE will both contribute to variation among chimaeric epiblasts. In the examples shown for both hypotheses, a high proportion of grey cells are allocated to the epiblast (C and F).

Initially, the two aggregated cell populations predominantly appear to occupy two hemispheres, while the ICM and TE are formed from inner and outer cells, respectively, so they form two concentric spheres. The geometry of these relationships led Falconer and Avery to reason that the composition of the ICM and TE would usually be similar in aggregation chimaeras. They proposed that most of the variation among chimaeric epiblasts (which produces the entire foetus and adult) would arise at the second allocation step, when ICM cells are allocated to either the epiblast or primitive endoderm (PrE), which forms a layer of cells between the epiblast and blastocoel cavity at the late blastocyst stage (Falconer and Avery, 1978). Their hypothesis is illustrated in Fig. 1A–C.

Falconer and Avery (1978) developed a mathematical model to support the hypothesis that most of the variation among epiblasts in chimaeric blastocysts, composed of ‘black’ and ‘white’ cell populations, arose by spatially constrained, geometrical sampling at the second allocation step. This assumed that the proportion of each cell population that was allocated to the epiblast would depend on the angle, at which the border between the epiblast and PrE intersected the boundary between the ‘black’ and ‘white’ domains in the chimaeric ICM. It further assumed that this angle would be random. This model predicted that the frequency distribution for the compositions of epiblasts of aggregation chimaeras would be flat across most of the range of percentage values but have peaks at 0% and 100% ‘white’ cells if two conditions were met. These conditions were that (1) approximately 50% of each cell population in the aggregate should be allocated to the ICM and (2) less than 50% of the ICM cells should be allocated to the epiblast.

One issue with Falconer and Avery's analysis is that their distributions of mouse adult coat pigmentation probably included non-chimaeric, technical failures. Analysis of foetal-stage chimaeras can distinguish between non-chimaeric conceptuses (non-chimaeric foetus and extraembryonic tissues) and non-chimaeric foetuses with chimaeric extraembryonic tissues. One such study showed that the distribution of foetal compositions from several pooled series of chimaeras was broad but not U-shaped, when non-chimaeric conceptuses (putative technical failures) were excluded but other non-chimaeric foetuses were included (West et al., 1995a). The appropriate distribution to model is, therefore, broad and possibly fairly flat but not U-shaped. Falconer and Avery's mathematical model primarily explains how a U-shaped frequency distribution could arise but their hypothesis can also produce flatter distributions. For example, no epiblasts with 0% or 100% ‘white’ cells would be produced if the ICM comprised 50% ‘white’ cells and more than 50% of the ICM cells were allocated to the epiblast.

Nevertheless, we have two concerns with Falconer and Avery's spatially constrained, geometrical allocation hypothesis. These stem from an improved understanding of how cells are allocated to the primary developmental lineages in mouse blastocysts (Johnson and McConnell, 2004; Saiz and Plusa, 2013; White et al., 2018). Early lineage tracing experiments showed that, on average, 58% of cells in non-chimaeric eight-cell embryos contributed to both the ICM and TE and the others contributed only to TE (Balakier and Pedersen, 1982). By the 16-cell stage in embryos, some cells are polarised outer cells (future TE cells) and others are apolar inner cells, which become ICM cells (Ziomek and Johnson, 1980; Johnson and Ziomek, 1981). At the next division, the inner cells normally produce only more inner cells. However, while some outer cells produce only more outer cells, others produce both inner and outer cells (Ziomek and Johnson, 1982). Our first concern is, therefore, that cellular heterogeneity may produce more variation among chimaeric ICMs than Falconer and Avery assumed, even if little cell mixing occurs in preimplantation chimaeras.

Our second criticism is that the geometrical mechanism proposed by Falconer and Avery (1978) for the second allocation event is very unlikely to be correct. This is because evidence now implies that the allocation of ICM cells to epiblast or PrE is driven by differences in gene expression, which precede differences in cell position within the ICM. In non-chimaeric embryos, specification of these two cell types is thought to begin by E3.25 (16–32 cells). Initially, future epiblast and PrE cells are finely intermixed within the ICM and form a stable ‘salt-and-pepper’ distribution of presumptive epiblast and presumptive PrE cells by about E3.5 (32–64 cells). The two cell types then sort-out at the late blastocyst stage, move to their final locations and complete differentiation by E4.5 (Chazaud et al., 2006; Plusa et al., 2008; Lanner and Rossant, 2010; Grabarek et al., 2012; Stephenson et al., 2012; Takaoka and Hamada, 2012; Saiz and Plusa, 2013; Chazaud and Yamanaka, 2016; Kang et al., 2017). This cell sorting is thought to involve a combination of cell movement, differential adhesion and selective apoptosis (Plusa et al., 2008; Saiz and Plusa, 2013). The evidence that specification of epiblast and PrE cells occurs in individual cells, rather than in spatially separated groups of cells provides strong evidence against the geometrical allocation mechanism, proposed by Falconer and Avery (1978) to explain the broad frequency distributions of coat pigmentation for adult chimaeras.

An alternative hypothesis is that both allocation steps (allocation of morula cells to ICM versus TE and allocation of ICM cells to PrE versus epiblast) contribute significantly to the variation among chimaeric epiblasts, both steps are at least partly stochastic in nature and the second allocation step does not depend on the geometrical constraints proposed by Falconer and Avery (1978). This stochastic, two-step hypothesis is consistent with an initially random distribution of epiblast and PrE precursors within the ICM (Fig. 1D–F). In chimaeric blastocysts, the percentage of each cell population initially allocated to the epiblast and PrE lineages may also be modified after the formation of nascent epiblast and PrE cells if selective apoptosis occurs.

Results from a preliminary attempt to test Falconer and Avery's hypothesis, by analysing the composition of mid-gestation chimaeras, were more consistent with a two-step hypothesis, with significant variation arising both before and during allocation of ICM cells to epiblast and PrE, but relatively few chimaeras were analysed (West et al., 1984).

The first aim of the present study was to analyse the composition of chimaeric blastocysts and a larger number of mid-gestation chimaeras, produced in several previously published studies, to characterise the variation among chimaeras at these stages and determine whether both the first and second allocation steps contributed to this variation. The second aim was to determine whether stochastic allocation of cells to different blastocyst lineages could explain the broad distribution of chimaeric epiblast compositions, without the type of geometrical mechanism that Falconer and Avery (1978) proposed for the second allocation step. For this we simulated two stochastic allocation steps in preimplantation chimaeras. We compared simulations that generated low versus higher levels of variation in composition among ICMs at the first allocation step and also compared the effects of producing different numbers of epiblast cells at the second allocation step.

## RESULTS

### Composition of different lineages in chimaeric blastocysts

**Fig. 2.**
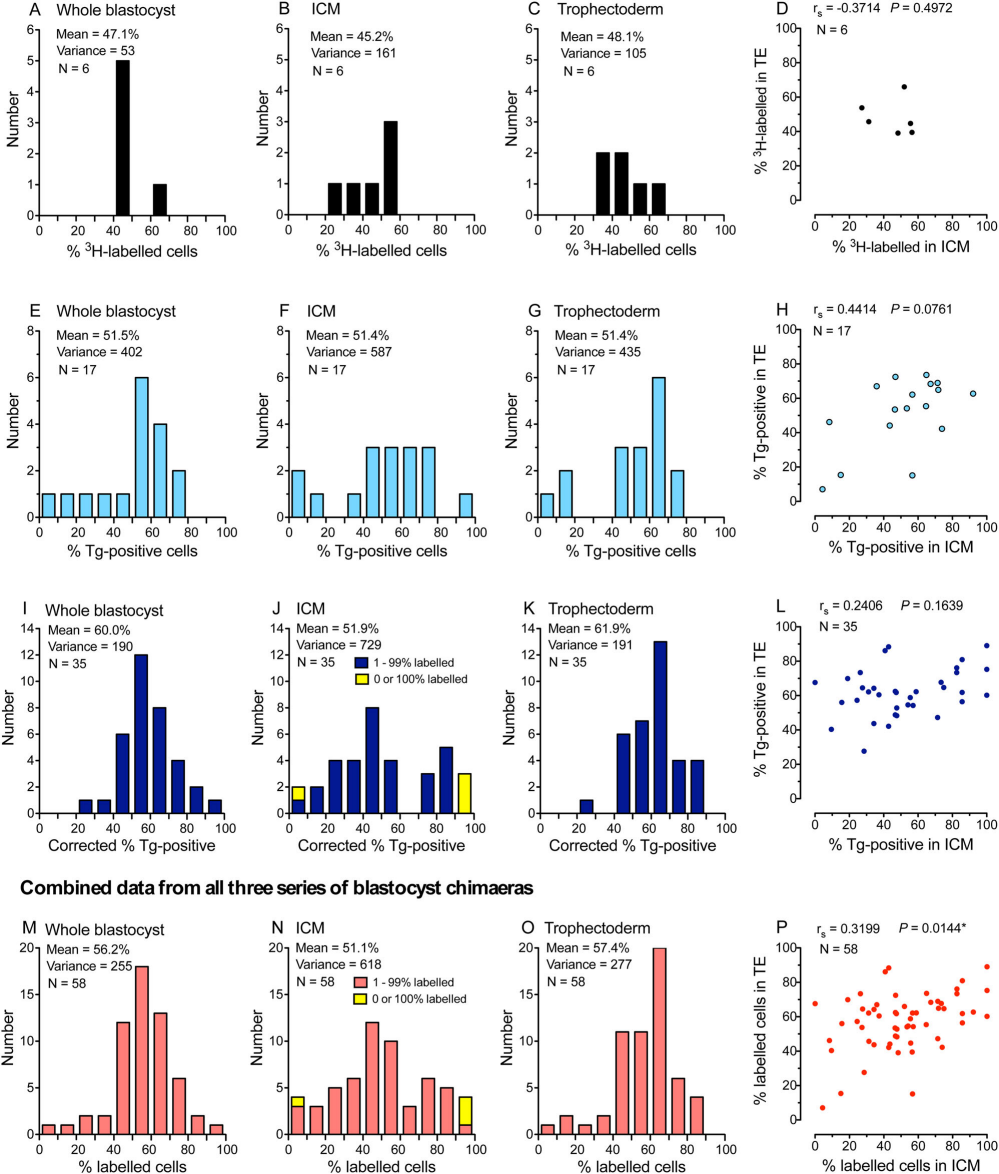
**Composition of mouse blastocyst chimaeras.** The percentage of labelled cells in three series of blastocyst chimaeras. (A–D) Six ^3^H-labelled chimaeric blastocysts in series Bl-^3^H. (E–H) 17 chimaeric blastocysts carrying a reiterated lineage marker (Tg) in series Bl-Tg1. (I–L) 35 chimaeric blastocysts carrying the Tg marker in series Bl-Tg2. (M–P) Data combined from all three series of chimaeric blastocysts. Frequency distributions are shown for the whole blastocyst (A,E,I,M), inner cell mass, ICM (B,F,J,N) and trophectoderm, TE (C,G,K,O). The correlation in percentage of labelled cells in the ICM and TE is shown in (D,H,L,P) with the Spearman correlation coefficient (r_s_) and *P*-value: *P<0.05.

Falconer and Avery's geometrical allocation hypothesis predicted that little variation arises before the second allocation step, so the extent of variation in composition of ICMs should be similar to that for whole blastocysts, whereas a stochastic, two-step hypothesis predicts that variation should be significantly greater among ICMs than whole blastocysts. We compared the composition of the whole blastocyst, ICM and TE in three published series of blastocyst chimaeras made by embryo aggregation, where the composition of the whole blastocysts were analysed. These studies are listed in Table S1, further details are given in the Materials and Methods and results are shown in Fig. 2 and Table S2.

Fig. 2 shows that there was significant variation in overall composition among individual blastocyst stage aggregation chimaeras (Fig. 2A,E,I,M), suggesting the possibility that the composition of chimaeric morulae varied before cells were allocated to the ICM or TE. If variation among chimaeric morulae before the first allocation step were negligible, the correlation in composition between the ICM and TE would be expected to be negative. However, the correlation for the pooled data was positive and weakly significant (Fig. 2P), supporting the suggestion that significant variation existed among chimaeric morulae before formation of the ICM. The source of this variation was not investigated but it could include biological variation between aggregated embryos and experimental variation that arises during the preimplantation stage of the chimaera production process (e.g. embryo damage or incomplete aggregation). Both of these types of variation would contribute to the normal variation among aggregation chimaeras but any technical errors in marker detection could cause additional variation and lead to overestimation of the variation among preimplantation stage chimaeras.

In each series, the variance in composition was lowest for the whole blastocyst, higher for the TE and highest for the ICM. This implies that the first allocation event increases the variance in the TE and ICM lineages. Together, the results from preimplantation chimaeras indicate that there is significant variation among chimaeric ICMs and this is likely to arise both before and during allocation of cells to the ICM and TE. However, these preimplantation stage chimaeras provided no information about the relationship between the epiblast and PrE, so we also investigated post-implantation stage chimaeras.

### Preliminary characterisation of eight series of E12.5 chimaeras

We analysed published data for the percentage of the GPI1A (glucose phosphate isomerase-1A) electrophoretic marker in mid-gestation *Gpi1^a/a^*↔*Gpi1^b/b^* aggregation chimaeras. Chimaeric tissues contained both GPI1A and GPI1B cells whereas non-chimaeric tissues contained only GPI1A or GPI1B cells. Foetuses and four extraembryonic tissues were analysed for the eight series of E12.5 chimaeras, listed in Table S1 (West and Flockhart, 1994; West et al., 1995b; Tang and West, 2001; MacKay et al., 2005). The foetus, amnion and yolk sac mesoderm (YSM) are all derived from the epiblast but the yolk sac endoderm (YSE) is from the PrE. In these experiments, placental GPI was almost entirely from the polar trophectoderm (pTE), because maternal GPI1 was all GPI1C, and so was excluded by electrophoresis (see the Materials and Methods), and other developmental lineages only produce about 4% of the mouse placenta (Rossant and Croy, 1985). Results for parietal endoderm samples (PrE lineage) were also available for four of the eight series of chimaeras but, as this tissue was not analysed in all the chimaeras, it was excluded from the preliminary characterisation. We analysed results for 285 E12.5 conceptuses, produced by embryo aggregation. There were 233 chimaeric conceptuses and 52 non-chimaeric conceptuses. The latter were considered separately from non-chimaeric samples from chimaeric conceptuses.

In the original publications, the eight series of E12.5 chimaeras were divided into four balanced and four unbalanced strain combinations according to the distributions of the percentage GPI1A in epiblast-derived samples (Tables S3 and S4). The frequency distribution for a specific sample type (e.g. amnion) from a series of E12.5 chimaeras was classified as balanced if the numbers of samples with <50% GPI1A did not differ significantly from the number with >50% GPI1A (West and Flockhart, 1994; West et al., 1995b). The series of chimaeras (and, therefore, that strain combination) was then classified as balanced or unbalanced according to the classification of the distribution for the foetus and other epiblast lineage samples. Compared to the balanced series of chimaeras (Table S3), the four unbalanced series had a lower proportion of epiblast-derived samples with >50% GPI1A (Table S4). In most cases, the balance of the YSE and placenta followed those of the epiblast-derived samples but there were a few exceptions (Tables S3 and S4). In all eight series, most placental samples had <25% or >75% GPI1A, so these placental distributions were considered ‘atypical’. Compared with the pooled balanced set of four chimaera series, the pooled unbalanced set had significantly more non-chimaeric conceptuses (Table S5) and more non-chimaeric samples from chimaeric conceptuses (Table S6). Moreover, fewer of the non-chimaeric samples were 100% GPI1A rather than 100% GPI1B (Table S6). As there were major differences between the balanced and unbalanced strain combinations, we analysed them separately.

Even for the balanced strain combinations, production of E12.5 chimaeras yielded 15 non-chimaeric conceptuses (non-chimaeric foetus, amnion, YSM, YSE and placenta) as well as 115 chimaeric conceptuses (Table S5). This shows that technical failure occurs during chimaera production and suggests that experimental variation that arises during chimaera production is likely to be significant among the chimaeric conceptuses.

### Characterisation of the frequency distributions for composition of E12.5 chimaeras

**Fig. 3.**
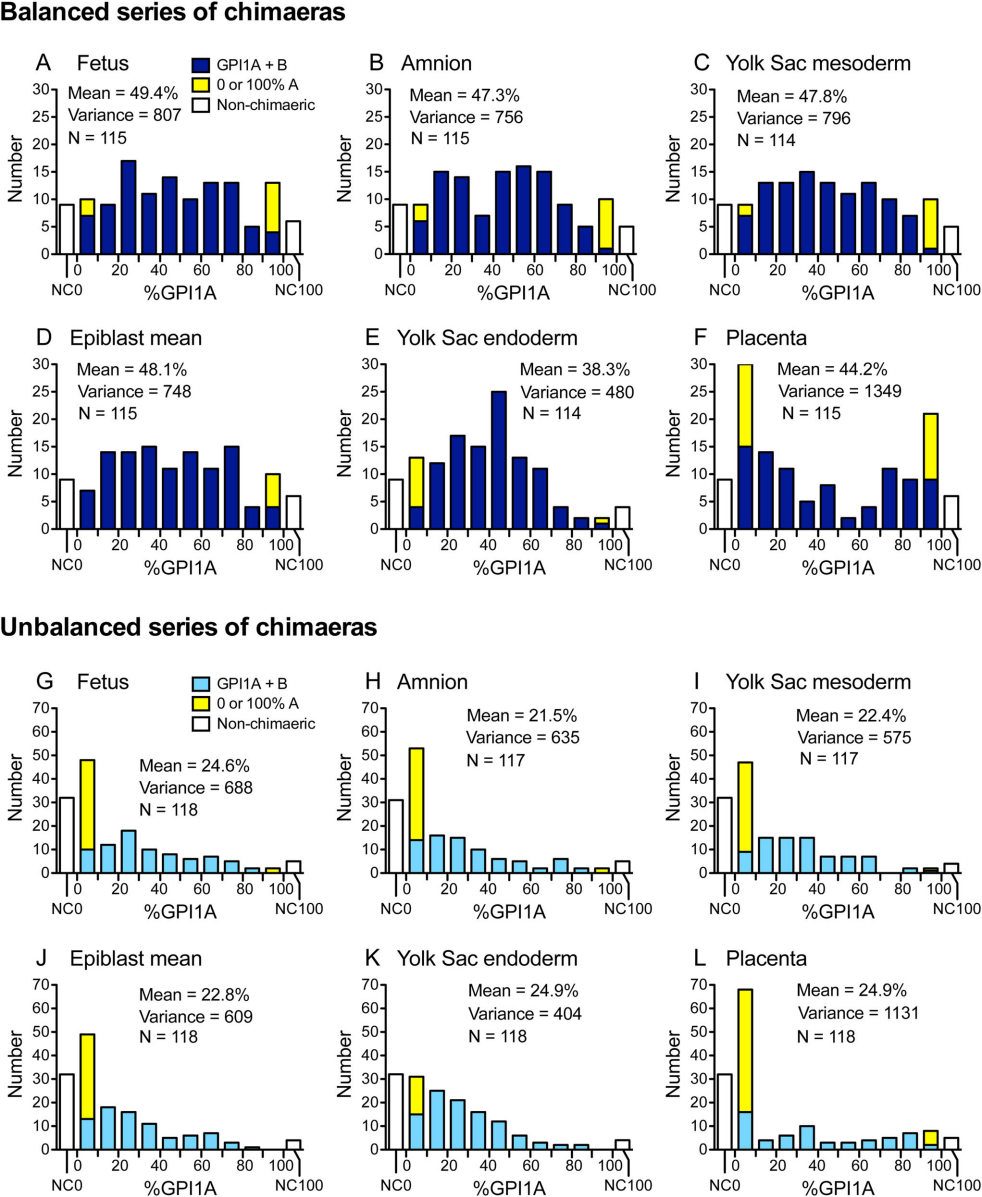
**Frequency distributions of the percentage of GPI1A in different samples from balanced and unbalanced series of E12.5 chimaeric conceptuses.** (A–F) Combined percentage GPI1A frequency distributions for four published balanced series of aggregation chimaeras: series XM, XP, PCT-VI and GMA. (G–L) Combined percentage GPI1A frequency distributions for four published unbalanced series of aggregation chimaeras: series XR, XN, PCT-V and GMB. Non-chimaeric conceptuses had 0% or 100% GPI1A in the foetus and all extraembryonic samples and are shown as white bars at the ends of the distributions. Non-chimaeric samples from chimaeric conceptuses are shown as yellow bars with the 0–10% GPI1A or 90–100% GPI1A group as appropriate. The mean, variance and number of samples (N), shown in the figures, exclude non-chimaeric conceptuses. See Table S1 for details of chimaera strain combinations and references to original publications. NC0, non-chimaeric conceptus with 0% GPI1A; NC100, non-chimaeric conceptus with 100% GPI1A.

Frequency distributions for the percentage of GPI1A in different samples from pooled balanced and pooled unbalanced series of chimaeras are shown in Fig. 3 and distributions for the eight individual series are shown separately in Figs S1 and S2. Non-chimaeric conceptuses (which are likely to be technical failures) are shown as white bars at the ends of the distributions and non-chimaeric samples (0 or 100% GPI1A) from chimaeric conceptuses are shown as yellow bars. This differs from how Falconer and Avery presented their results for coat pigmentation in adult chimaeras, as they did not distinguish between non-chimaeric mice and chimaeras with non-chimaeric coat pigmentation (Falconer and Avery, 1978).

The percentage GPI1A distributions of the epiblast-derived samples (foetus, amnion and YSM) from the pooled balanced series were relatively flat (Fig. 3A–D) but the YSE distribution appeared to be skewed and more hump-shaped (Fig. 3E). Parietal endoderm distributions appeared to be similar to those of the YSE but they were only analysed for two balanced and two unbalanced series of chimaeras (Figs S1 and S2). In contrast, the placentas from the pooled balanced series of chimaeras showed a broad U-shaped distribution (Fig. 3F). All samples from the pooled unbalanced series were highly skewed towards a low percentage GPI1A (Fig. 3G–L). This analysis confirmed that the distributions for the compositions of the foetus and other epiblast derivatives from the balanced series of chimaeras were much flatter than the distributions reported for X-inactivation mosaics (Falconer and Avery, 1978; West et al., 1984) and did not show the broad U-shaped distribution described by Falconer and Avery (1978) for coat pigmentation in adult chimaeras.

### Relationships within and between epiblast and primitive endoderm lineages in E12.5 chimaeric conceptuses

**Fig. 4.**
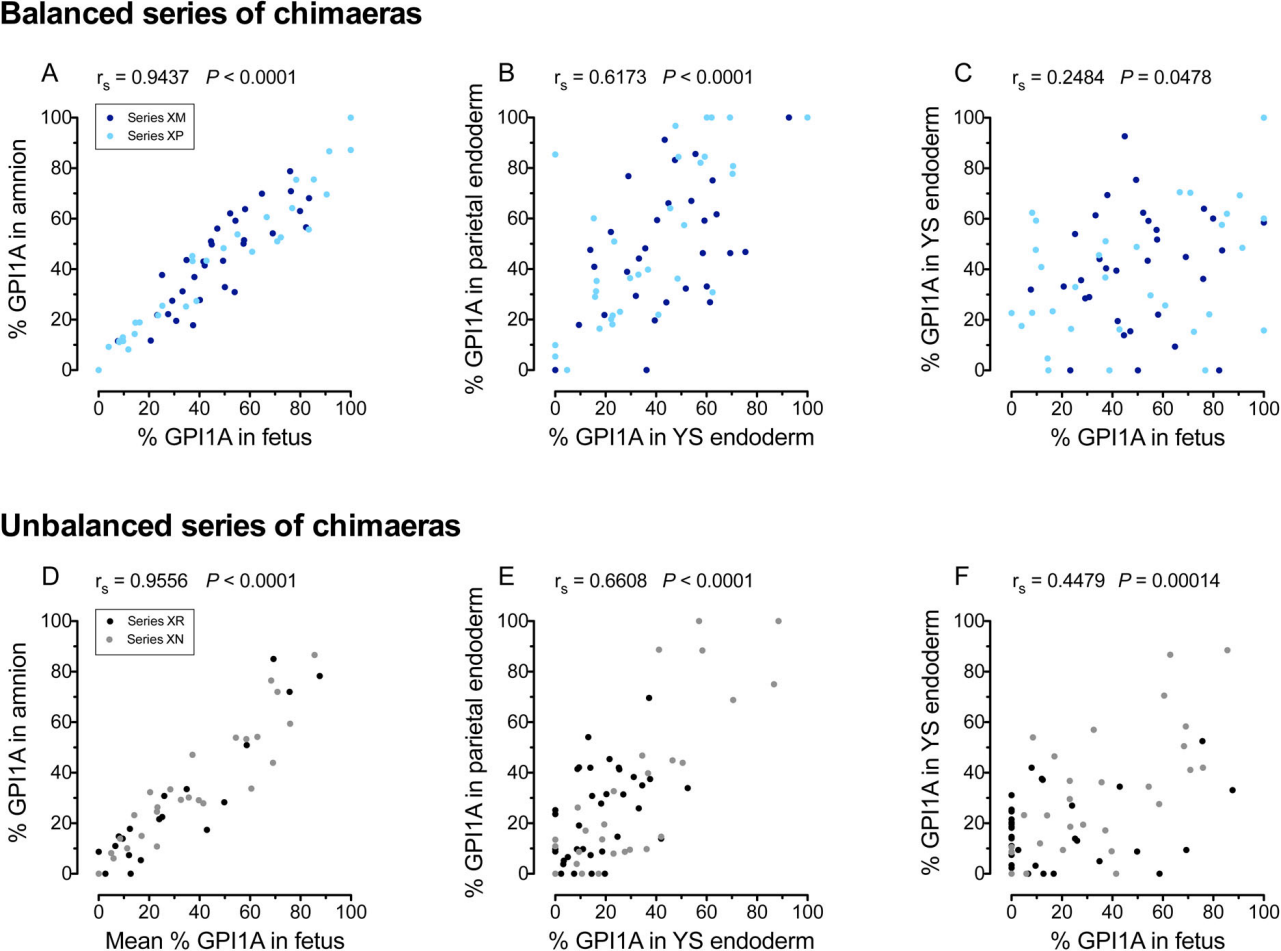
**Correlations in the percentage of GPI1A between different samples from E12.5 chimaeras.** Correlations between representative samples from the epiblast lineage (foetus and amnion) and the primitive endoderm lineage (yolk sac endoderm and parietal endoderm) from two published balanced series of chimaeras (A–C) and two published unbalanced series of chimaeras (D–F). Spearman correlation coefficients (r_s_) and *P*-values are shown on the graph. See Tables S7 and S8 for Spearman correlations between other samples.

If the second allocation step, involving the segregation of the epiblast and PrE lineages, caused variation among chimaeric epiblasts, samples within either of these lineages should show stronger positive correlations in composition (higher correlation coefficients) than samples derived from different lineages. Fig. 4 shows that this prediction was borne out for representative epiblast samples (foetus and amnion) and the two PrE-derived samples from the four series of chimaeras for which the parietal endoderm was analysed (series XM, XP, XR and XN). For each of these four individual series of chimaeras, correlations were strongest for pairs of samples within the epiblast lineage (foetus, amnion and YSM) but they were also stronger within the PrE lineage (YSE and parietal endoderm samples) than between any epiblast-derived sample and either of the PrE-derived samples (Tables S7 and S8). This implies that the second allocation step is a significant source of variation among chimaeric epiblasts.

If there were very little variation in composition among ICMs before the second allocation step, the correlation between the epiblast-derived samples and PrE-derivatives would be expected to be negative. As none of those correlations in the eight series of E12.5 chimaeras was negative (Fig. 4C,F; Tables S7 and S8), this implies that significant variation existed among ICMs. This variation must have arisen either before or during the first allocation step when cells were allocated to the ICM or TE. This result is consistent with the analysis of preimplantation stage chimaeras, described above.

### Computer simulation of a stochastic two-step hypothesis

We designed two blastocyst chimaera allocation models (A and B) to simulate low and higher levels of variation, respectively, at the first allocation step. Each model comprised three series of simulations (A1–A3 and B1–B3), which simulated different numbers of ICM cells allocated to the epiblast versus the PrE at the second allocation step. For each of these six series, ten sets of 1000 chimaeric embryos with ‘black’ and ‘white’ cells were simulated. The second allocation step (formation of epiblast and PrE) contributed variation to the epiblast composition in both models A and B but it was not constrained spatially in the way that was proposed by Falconer and Avery (1978). As explained in the Materials and Methods, we sought to limit the number of variables in the simulations and use biologically realistic values. For simplicity, all the simulated chimaeric blastocysts had 50% black cells overall and neither cell death nor differences in cell proliferation was simulated. Although the proportion of inner cells in model B was increased at each round of cell divisions (from 5/16 to 12/32 to 28/64) we did not attempt to simulate the reported variability in this proportion among embryos at the same stage (see the Materials and Methods).

At the time of the second allocation step it was assumed that there were always 28 ICM cells and 36 TE cells in both models A and B. It is unclear whether more mouse ICM cells are allocated to become prospective epiblast or prospective PrE cells (see the Materials and Methods). Thus, to produce variation at the second allocation step, different numbers of simulated ICM cells were allocated to epiblast and PrE in the three series of simulations compared for each model (ten epiblast cells in series A1 and B1, 14 in A2 and B2 and 18 in A3 and B3).

**Fig. 5.**
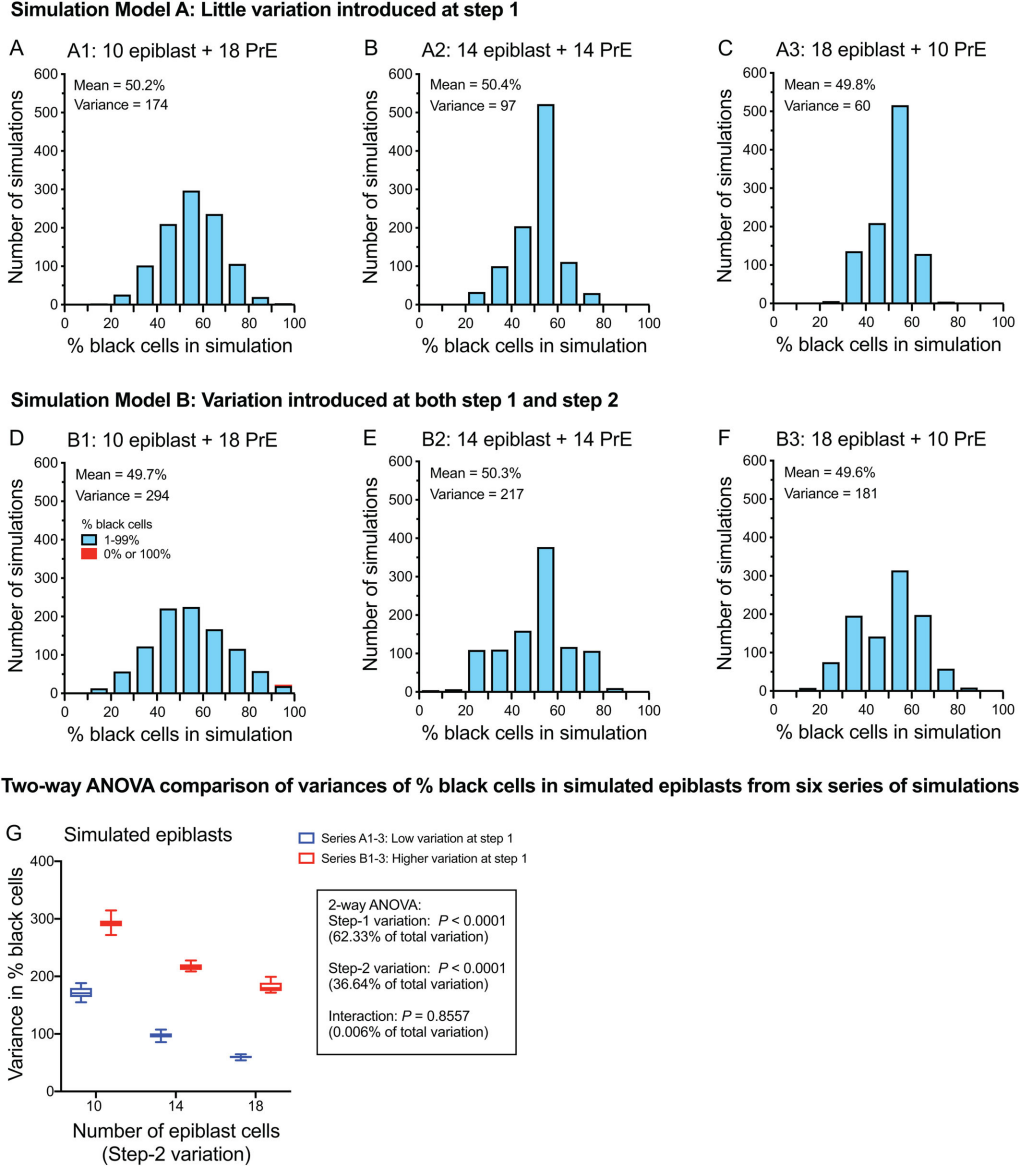
**Variation in the percentage of black cells in simulated chimaeric blastocyst epiblasts in six series of simulations.** (A–F) Frequency distributions for the percentage of black cells in simulated epiblasts from a representative set of 1000 simulated 64-cell, chimaeric blastocysts for each of six series of simulations. (The representative set of simulations had an epiblast variance that was closest to the mean epiblast variance for the ten sets in that series.) No cell death was simulated and all simulated blastocysts had 50% black cells overall, 28 ICM cells and 36 TE cells. The numbers of epiblast and PrE cells varied among series A1–A3 and among series B1–B3. (A–C) Results for series A1–A3 from simulation model A, in which little variation was introduced at allocation step 1, when cells were allocated to the TE and ICM (all ICMs had 46.4–53.6% black cells) but more variation was introduced at allocation step 2, when ICM cells were allocated to the epiblast and PrE. (A) Series A1 with ten epiblast cells and 18 PrE cells. (B) Series A2 with 14 epiblast cells and 14 PrE cells. (C) Series A3 with 18 epiblast cells and ten PrE cells. (D–F) Results for series B1–B3 from simulation model B, in which variation was introduced at both step 1 and step 2. (D) Series B1 with ten epiblast cells and 18 PrE cells. (E) Series B2 with 14 epiblast cells and 14 PrE cells. (F) Series B3 with 18 epiblast cells and ten PrE cells. The mean percentage of black cells and its variance are shown in the figure. (G) Comparison of the variance in the percentage of black cells in the epiblast for all ten sets of simulations for each of the six series of simulations by two-way ANOVA. Step-1 variation compares series A versus B (low and higher variation at step 1) and step-2 variation compares series 1, 2 and 3 (different numbers of epiblast cells at step 2). Box and whisker plots show the median (horizontal line within the box), upper and lower quartiles (top and bottom of boxes) and the minimum and maximum values (ends of whiskers).

Fig. 5A–F shows frequency distributions for the percentage of black cells in the epiblast for a representative set of each of the six series of simulations. As expected, the percentage of black cells in the epiblast lineage was more variable in model B, where significant variation was introduced at both steps 1 and 2, than in model A, where significant variation was introduced only at step 2 (compare spread of distributions and variances in Fig. 5A–C with Fig. 5D–F). For each model, more epiblast variation occurred when fewer ICM cells were allocated to the epiblast lineage (compare the spread of the distribution and variance in Fig. 5A to Fig. 5B and C, and compare Fig. 5D to Fig. 5E and F). Thus, the epiblast distribution for simulation series B1 had the broadest distribution with the largest variance (Fig. 5D). This was confirmed by two-way analysis of variance (ANOVA), using results from all 60 sets of simulations (Fig. 5G). Frequency distributions for other simulated blastocyst lineages for these six representative sets of simulations are shown in Figs S3 and S4 and results for all 60 sets are summarised in Figs S5 and S6.

## DISCUSSION

### Origin of variation among chimaeric epiblasts

Our analysis of the balanced series of E12.5 chimaeras showed that frequency distributions of epiblast derivatives were consistent with fairly flat distributions but not the U-shaped distributions reported for the percentages of adult coat pigmentation (Falconer and Avery, 1978). Falconer and Avery reported U-shaped distributions for individual series of chimaeras, so they are unlikely to be composite distributions of two genotype combinations that were unbalanced in opposite directions. As suggested in the Introduction, the U-shaped coat pigmentation distributions are more likely to have resulted from inclusion of non-chimaeric mice that developed from non-chimaeric conceptuses, many of which probably arose from technical failures.

Falconer and Avery assumed that there is initially little variation in composition among chimaeric aggregates, little variation arises when morula cells are allocated to the ICM or TE and that almost all the variation arises when ICM cells are allocated to the epiblast or PrE. However, this is not supported by results reported here. Evidence from preimplantation stage chimaeras suggests that some variation already exists among aggregates before segregation of the ICM and TE. This would include both biological variation between aggregated embryos and experimental variation introduced by the chimaera production procedures. Even if this initial variation is overestimated for technical reasons, combined evidence from studies with preimplantation and post-implantation stage chimaeras implies that significant additional variation arises both when cells are allocated to the ICM or TE and when ICM cells are allocated to the epiblast or PrE.

The computer simulations should be interpreted with caution, because several potential sources of biological variability were not included. For example, the simulations did not allow for biological differences between the aggregated embryos, in developmental stage, rate of cell proliferation or frequency of cell death, nor did they allow for experimental variation caused by the chimaera production procedure. Despite our biological evidence to the contrary, for simplicity, it was assumed that there was no difference in composition among chimaeras before cells were allocated to the ICM and TE and, as cell loss was not simulated, each simulated chimaeric embryo had 50% black cells, overall. Although the proportion of simulated inner cells was increased at each round of cell divisions in model B (see the Materials and Methods), the simulations did not allow for variation in inner cell numbers within a stage, although such variation is known to exist (Handyside, 1978, 1981). Thus, the simulations oversimplified the biology and probably underestimated the variation among chimaeric epiblasts.

Despite these conservative simplifications, the computer simulations confirmed that, in principle, a fairly broad frequency distribution of simulated chimaeric epiblast compositions could be produced by two stochastic allocation steps, without the type of spatial constraints proposed by Falconer and Avery (1978). This simulated variation arose in the absence of any variation before allocation step 1 because all the simulated chimaeric blastocysts had 50% black cells overall. Nevertheless, the simulated epiblast distributions were less variable than the epiblast samples from E12.5 chimaeras. This may be both because the simulations underestimated the extent of the biological and experimental variation (as discussed above) and because subsequent allocation steps increase the variation between the blastocyst stage and mid-gestation.

Together, our analyses of chimaera results and computer simulations showed that stochastic allocation of cells to different blastocyst lineages could cause much of the variation in chimaeric epiblast composition, without invoking the type of geometrical allocation mechanism proposed by Falconer and Avery (1978). Later allocation events will contribute to variation among post-implantation epiblast derivatives in both chimaeras and X-inactivation mosaics. The wide variation in compositions of foetal chimaeras is probably explained by a combination of allocation events in the blastocyst and at later stages.

### Origin of variation among chimaeric placentas

The U-shaped frequency distribution reported for the composition of chimaeric placentas, both here and previously described (West et al., 1995a), differs markedly from those of samples produced by the epiblast or PrE lineages but is similar to that originally reported for coat colours of adult chimaeras (Falconer and Avery, 1978). Our simple simulation of allocation events in the chimaeric blastocyst does not explain this distribution and the simulated TE distributions were not bimodal or U-shaped. The placenta is largely derived from the pTE, which forms a hemisphere of TE adjacent to the ICM in the early blastocyst. As the TE cells are likely to remain relatively unmixed in chimaeric blastocysts (Garner and McLaren, 1974), the border between the pTE and mTE may intersect the main boundary between the labelled and unlabelled cells in the chimaeric TE at any angle. This type of geometrical allocation of TE cells to pTE or mTE resembles the geometrical allocation of ICM cells proposed by Falconer and Avery (1978) to explain the apparent U-shaped distribution of adult coat colours. However, we have not investigated whether geometrical allocation would be sufficient to produce the U-shaped placental distribution.

**Fig. 6.**
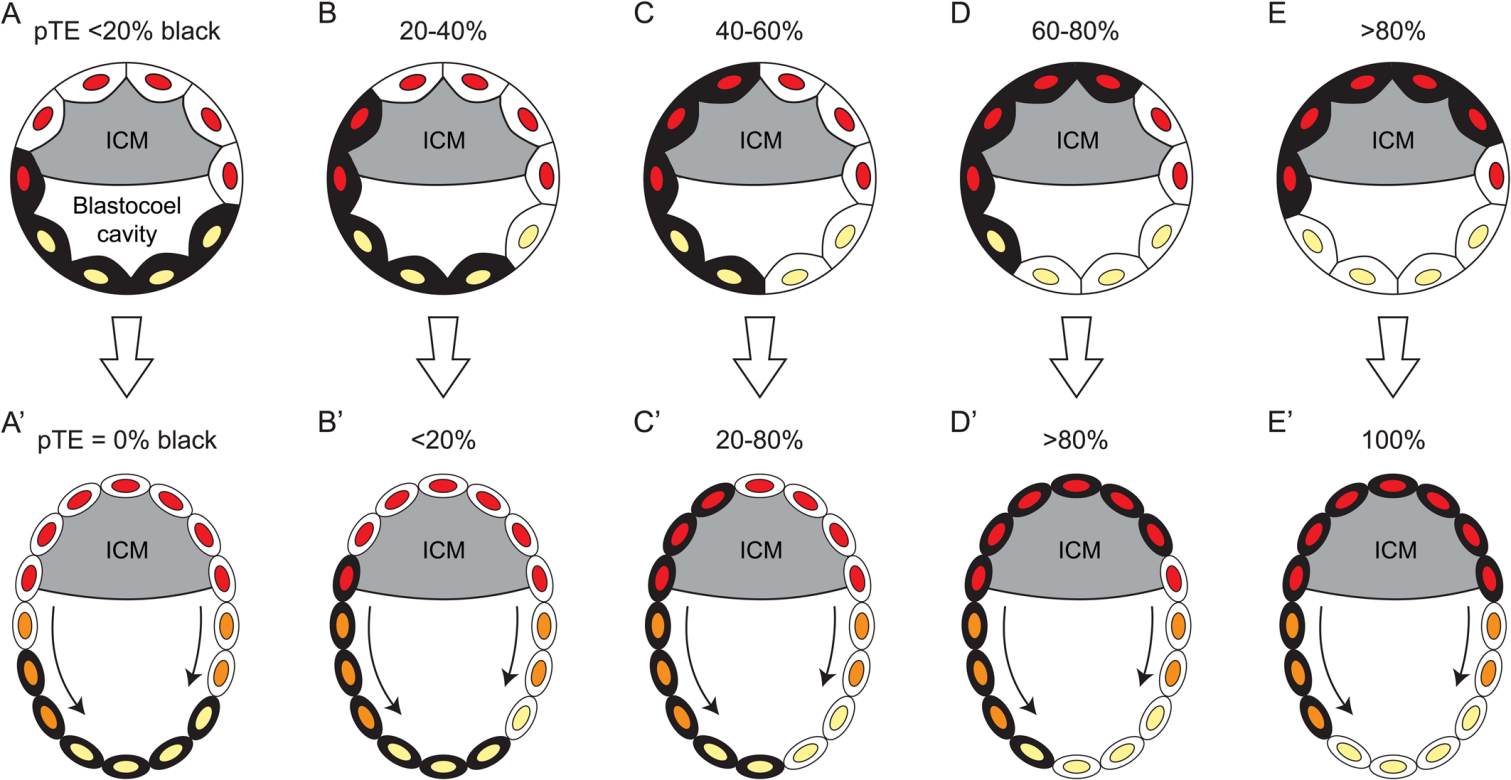
**Diagram showing how the frequency of chimaeric blastocysts with non-chimaeric polar trophectoderm lineages might increase during development.** (A–E) Diagram of five chimaeras at the early blastocyst stage, each with two cell populations of trophectoderm (TE) cells, shaded black and white, respectively. TE nuclei are shaded according to their lineage: red, polar trophectoderm (pTE); yellow, mural trophectoderm (mTE). The inner cell mass (ICM) is shown as a grey shaded area but individual ICM cells are not shown. The five chimaeras are shown with different hypothetical initial percentages of black cells in the pTE. In the early blastocyst, the pTE cells overlie the ICM and the mTE cells are adjacent to the blastocoel cavity and do not contact the ICM. The black and white TE cells are likely to remain relatively unmixed (Garner and McLaren, 1974), so the percentage of black TE cells allocated to the pTE will depend on the angle at which the boundary between the pTE and mTE intersects the boundary between the black and white TE cell populations. (A′–E′) During blastocyst growth, the mTE cells stop dividing but the pTE cells proliferate and some move to form new mTE cells (orange nuclei) (Copp, 1978, 1979; Gardner, 2000). This might deplete the minority pTE cell population and so increase the frequency of non-chimaeric polar trophectoderm lineages, thereby increasing the variance of the percentage black pTE cells in a series of chimaeras. Movement of cells from the pTE and mTE is shown at both sides of the blastocysts in the diagrams but such movement may not be symmetrical (Gardner, 2000).

The flow of TE cells, moving from the pTE to the mTE (Copp, 1978, 1979; Gardner, 2000), could also help produce a U-shaped distribution. As this flow is unidirectional, it could sometimes result in the loss of one pTE cell population to the mTE in a chimaeric blastocyst, thereby increasing the number of non-chimaeric pTEs (Fig. 6). Thus, the U-shaped frequency distributions observed for the composition of chimaeric placentas might be explained by a combination of geometrical allocation of TE cells to the pTE and the subsequent flow of pTE cells to the mTE.

### Conclusions

Our analysis of chimaera composition data implies that both allocation of morula cells to the ICM or TE and allocation of ICM cells to the epiblast or PrE significantly increase the variation among chimaeric epiblasts. It also suggests that some heterogeneity exists among aggregates, even before the first allocation step. Moreover, computer simulation results were consistent with the possibility that significant variation among chimaeric epiblasts could arise by two stochastic allocation steps. Together, these analyses show that much of the variation in chimaeric epiblasts can be explained by stochastic allocation of cells to different blastocyst lineages without the type of spatial constraints proposed by Falconer and Avery (1978). Allocation of cells to different lineages at blastocyst and later stages probably explains the wide variation in compositions of foetal chimaeras. We also suggest that the U-shaped frequency distributions, characteristic of placentas from balanced series of mid-gestation chimaeras, might be explained by geometrical allocation of TE cells to the pTE and/or the subsequent movement of some TE cells from the pTE to the mTE.

## MATERIALS AND METHODS

### Blastocyst chimaeras

Results from three published series of chimaeric blastocysts were analysed. Series Bl-^3^H was a series of six eight-cell ^3^H-thymidine-labelled↔eight-cell unlabelled chimaeric blastocysts analysed by autoradiography at approximately E4.3 (Garner and McLaren, 1974). The percentage of ^3^H-thymidine-labelled cells in the whole blastocyst was calculated as a weighted mean, by weighting the published percentage ICM and TE values by the published numbers of ICM and TE cells.

Chimaeric blastocysts in two other published series were produced with embryos that were hemizygous for the *TgN(Hbb-b1)83Clo* reiterated marker transgene (Lo, 1983; Lo et al., 1987), here abbreviated to *Tg* (Table S1). Those in the series designated Bl-Tg1 were produced as a control series (originally called series CeB) by aggregating eight-cell, hemizygous *Tg*/- embryos with eight-cell, non-transgenic (−/−) embryos at E2.5 (Everett and West, 1996). Aggregates were cultured for a further 30 h until 90 h post coitum (p.c.) and analysed at approximately E3.8. Chimaeric blastocysts in the series designated Bl-Tg2 were produced as controls for a different study and were originally called series PCT-III or series S2n↔*S2n (Tang et al., 2000). Instead of aggregating whole embryos at E2.5, each chimaera in series Bl-Tg2 was made by aggregating half of a two-cell *Tg*/− embryo with half of a two-cell −/− embryo, and the aggregates were cultured for 3 days until E4.5. The marked cells were identified by DNA *in situ* hybridisation to the reiterated transgene on serial sections of blastocysts. For both series Bl-Tg1 and Bl-Tg2, all cells were scored as positive or negative and the percentage of Tg-positive cells was corrected using control data from *Tg*/−↔*Tg*/− controls (Everett and West, 1996; Tang et al., 2000). For series Bl-Tg2, the corrected percentage of Tg-positive cells in the whole TE was calculated as the weighted mean of pTE and mTE values and the corrected percentage of Tg-positive cells in the whole blastocyst was calculated as the weighted mean of ICM, pTE and mTE, using the cell numbers in ICM, pTE and mTE. Data were incomplete for three of the 38 chimaeric blastocysts in series Bl-Tg2, so results were only analysed for the other 35 chimaeras.

### E12.5 chimaeras

Data from eight series of E12.5 aggregation chimaeras (XM, XN, XP, XR, PCT-V, PCT-VI, GMA and GMB) from four published studies (West and Flockhart, 1994; West et al., 1995b; Tang and West, 2001; MacKay et al., 2005) were analysed in the present study. The strain combinations are shown in Table S1; other details are given in the original papers and are also summarised in Tang et al. (2018). The chimaeras were produced by aggregating one *Gpi1^a/a^* and one *Gpi1^b/b^* embryo and the aggregates were transferred to *Gpi1^c/c^* recipient females. Quantitative glucose phosphate isomerase (GPI) electrophoresis was used to estimate the contribution of *Gpi1^a/a^* and *Gpi1^b/b^* cells to different samples from the percentages of GPI1A and GPI1B allozymes. Maternal contamination or contribution to the placenta was identified as a separate GPI1C allozyme (*Gpi1^c/c^* cells) and excluded. Three dead conceptuses (one each in series XM, XN and GMB), one conceptus in series XR that was fused to a mole and twelve conceptuses that either shared extraembryonic membranes or had fused placentas (two each in series PCT-V and PCT-VI and four each in series XM and XR) were excluded from the analysis. Of the remaining 285 conceptuses, 233 were chimaeric and 52 were non-chimaeric in all of the samples analysed. Some technical losses occurred during analysis so data were incomplete for a few conceptuses.

### Computer simulation of chimaeric blastocyst

When designing the computer simulations, our aims were to restrict the number of variables to a manageable number and use values that were biologically plausible. Two simulation models of chimaeric blastocysts composed of ‘black’ and ‘white’ cells were developed to model how variation in the epiblast of chimaeric blastocysts could arise in two allocation steps without the type of geometrical sampling that was proposed by Falconer and Avery (1978). Variation in the percentage of black cells in each lineage was introduced at the first allocation step (formation of ICM and TE) and the second allocation step (formation of epiblast and PrE).

In simulation model A, the first allocation step introduced little variation. In this model, all 64-cell chimaeric blastocysts contained 28 ICM cells and 36 TE cells. Overall there were 50% black cells and 13, 14 or 15 of the ICM cells were black cells so the percentage of black cells in the ICM only varied from 46.4–53.6%.

In simulation model B, more variation was introduced at the first allocation step. A simulated eight-cell black embryo was aggregated with a simulated eight-cell white embryo to form an aggregate of 16 cells, which all underwent two rounds of cell divisions, without cell loss, to produce a 64-cell chimaeric blastocyst. As a chimaeric aggregate has twice the normal number of cells, it will have some inner cells from the outset and the simulation assumed that all aggregates initially had five inner and 11 outer cells (31.3% inner cells). In most biological aggregates, both embryos will contribute to the inner cells, so at least one black and one white inner cell were included in the inner cells of the simulated 16-cell aggregates. Three other cells were allocated randomly to the inner lineage and the remainder became outer cells.

For non-chimaeric mouse embryos, it has been reported that there are typically four to seven inner cells at the 16-cell stage, and the percentage of inner cells rises from 25–44% to about 43–48% by the 32-cell stage (Handyside, 1978, 1981). Guided by the biological data, the proportion of inner cells was increased from 5/16 over the next two rounds of cell divisions in model B simulations. After the first round of cell divisions, simulated 32-cell chimaeras had 12 inner and 20 outer cells (37.5% inner cells) and after the next round, simulated 64-cell chimaeras had 28 (43.8%) inner cells (ICM cells) and 36 outer cells (TE cells). At each division, all simulated inner cells divided symmetrically (conservatively) to produce two inner cells. In contrast, some outer cells divided symmetrically to produce two outer cells, while others divided asymmetrically (differentiatively) to produce one inner and one outer cell. Black and white outer cells were selected randomly for asymmetrical division.

In both simulation models, additional variation was introduced at the second allocation step, by simulating random allocation of black and white ICM cells to the epiblast (with unallocated ICM cells becoming PrE) in 64-cell stage chimaeric blastocysts. This is equivalent to the 32-cell stage in non-chimaeras, which is approximately when presumptive epiblast and PrE cells become specified (Plusa et al., 2008; Kang et al., 2017). The proportion of ICM cells that become epiblast cells remains unclear. An early report concluded that ICMs of non-chimaeric E4.5 embryos contained 37% epiblast cells and 63% PrE cells (Gardner and Rossant, 1979). However, a later report of gene expression indicated that approximately 55% of ICM cells in E3.75 embryos were specified as epiblast, 40% as PrE and 5% appeared to be still unspecified (Krawchuk et al., 2013). In agreement with Gardner and Rossant (1979), Saiz et al. (2016) reported that, in blastocysts with over 100 cells and less than 5% unspecified ICM cells, approximately 40% of the specified ICM cells were specified as presumptive epiblast and about 60% as presumptive PrE cells. However, earlier blastocysts had more unspecified ICM cells and, at the earliest stage analysed (32–64 cell stage) there did not appear to be fewer presumptive epiblast cells than presumptive PrE cells (Fig. S1b in Saiz et al., 2016). These different results could be explained if the frequencies of newly specified epiblast and PrE were initially similar but variable and were then modified to produce a final value of approximately 40% epiblast cells. The secondary modifications could involve modulation of the later specification events in older blastocysts (Saiz et al., 2016) and/or differential levels of cell death among presumptive epiblast and PrE cells, during cell sorting between E3.75 and E4.5. As far as we know, this has not been investigated.

As it is unclear whether more epiblast or PrE founder cells are produced, different numbers (10, 14 or 18) of the 28 simulated ICM cells were allocated to epiblast, in the three series of simulations compared for each model. We did not simulate cell death but the effects of unequal levels of death among newly specified epiblast and PrE cells is likely to be similar to unequal cell allocation of ICM cells to these two lineages and this was simulated.

The simulation was written with the programming language Python. For each of the six series of simulations, ten sets of 1000 chimaeric embryos were simulated. In the simulation code, a set of 1000 simulated chimaeras is referred to as a ‘run’.

### Statistics

The chimaera data were published previously so sample sizes were those used in the earlier studies. The choice of parametric or non-parametric tests was guided, in part, by normality tests. GraphPad Prism (GraphPad Software Inc., La Jolla, CA, USA) was used for most statistical tests including Fisher's exact test, Spearman correlation test, Student's *t*-test and two-way ANOVA. Online statistical calculator, http://vassarstats.net/index.html, was used for continuity corrected chi square goodness-of-fit tests and http://graphpad.com/quickcalcs/chisquared1.cfm, was used for uncorrected chi square goodness-of-fit tests.

## Supplementary Material

Supplementary information
