# Supplementary material for: Re-evaluation of the causes of variation among mouse aggregation chimaeras
Source: Biol Open. 2019 May 15;8(5):bio042804. doi: 10.1242/bio.042804 (PMC6550066; doi:10.1242/bio.042804)
Supplement: Supplementary information [file biolopen-8-042804-s1.pdf]

Table S1. Strain combinations used for blastocyst chimaeras and E12.5 chimaeras.

Chimaera series	Strain combination*	Original reference
**Blastocyst chimaeras:**		
Bl-^{3}H	8-cell ^{3}H-labelled Q strain↔8-cell unlabelled Q strain	Reference 1
Bl-Tg1	8-cell (C57BL×CBA)F2↔8-cell [(C57BL×CBA)F1 × TGB]	Series CeB in ref. 2
Bl-Tg2	½ 2-cell (C57BL×CBA)F2↔½ 2-cell [(C57BL×CBA)F1 × TGB]	S2n↔*S2n in ref. 3
**E12.5 chimaeras: balanced strain combinations (8-cell *Gpi1*$^{a/a}$↔8-cell *Gpi1*$^{b/b}$)**		
XM	(BC × BALB/c)F2↔(C57BL × CBA)F2	Series XM in ref. 4
XP	[(BC x BALB/c)F1 x BALB/c]↔(C57BL × CBA)F2	Series XP in ref. 5
PCT-VI	(BALB/c × A/J)F2↔[(C57BL × CBA)F1 × TGB]	Series PCT-VI in ref. 6
GMA	(BALB/c × A/J)F2↔[(C57BL × CBA)F1 × TP6.3]	Series GMA in ref. 7
**E12.5 chimaeras: unbalanced strain combinations (8-cell *Gpi1*$^{a/a}$↔8-cell *Gpi1*$^{b/b}$)**		
XR	(BALB/c × BALB/c)↔(C57BL × CBA)F2	Series XR in ref. 4
XN	[BALB/c × (BC × BALB/c)F1]↔(C57BL × CBA)F2	Series XN in ref. 5
PCT-V	(BALB/c × BALB/c)↔[(C57BL × CBA)F1 × TGB]	Series PCT-V in ref. 6
GMB	(BALB/c × BALB/c)↔[(C57BL × CBA)F1 × TP6.3]	Series GMB in ref. 7

*The female strain is shown first for all crosses.

Abbreviations: BALB/c, BALB/c/Eumm or BALB/c/OlaHsd; BC, C57BL/Ola.AKR-*Gpi1*^{a},*Tyr*^{c}/Ws; C57BL, C57BL/OlaHsd (derived from the British C57BL/Gn line and probably not completely identical with C57BL/6); CBA, CBA/Ca; TGB, stock carrying *TgN(Hbb-b1)83Clo* marker transgene; TP6.3, Stock carrying *TgTP6.3* tauGFP marker transgene

References

1. Garner, W. and McLaren, A. (1974). Cell distribution in chimaeric mouse embryos before implantation. *J. Embryol. Exp. Morphol.* 32, 495-503.
2. Everett, C. A. and West, J. D. (1996). The influence of ploidy on the distribution of cells in chimaeric mouse blastocysts. *Zygote* 4, 59-66.
3. Tang, P.-C., Ritchie, W. A., Wilmut, I. and West, J. D. (2000). The effects of cell size and ploidy on cell allocation in mouse chimaeric blastocysts. *Zygote* 8, 33-43.
4. West, J. D. and Flockhart, J. H. (1994). Genotypically unbalanced diploid↔diploid foetal mouse chimaeras: possible relevance to human confined mosaicism. *Genet Res* 63, 87-99.
5. West, J. D., Flockhart, J. H. and Kissenpfennig, A. (1995). A maternal genetic effect on the composition of mouse aggregation chimaeras. *Genet Res* 65, 29-40.
6. Tang, P.-C. and West, J. D. (2001). Size regulation does not cause the composition of mouse chimaeras to become unbalanced. *Int. J. Dev. Biol.* 45, 583-590.
7. MacKay, G. E., Keighren, M. A., Wilson, L., Pratt, T., Flockhart, J. H., Mason, J. O., Price, D. J. and West, J. D. (2005). Evaluation of the mouse *TgTP6.3* tauGFP transgene as a lineage marker in chimeras. *J. Anat* 206, 79-92.

Table S2. Cell numbers in different series of blastocyst chimaeras.

	Chimaeric blastocyst series		
	Bl-^{3}H	Bl-Tg1	Bl-Tg2
Embryos aggregated	8-cell↔8-cell	8-cell↔8-cell	½ 2-cell↔½ 2-cell
Culture period	E1.5 to E4.3	E2.5 to E3.8	E1.5 to E4.5
Number of blastocysts	6	17	35
Cell number (mean±95% CI)			
Total	64.0±7.7	116.2±18.1	102.3±8.3
Inner Cell Mass (ICM)	21.5±3.8	40.8±7.6	18.5±3.0
Trophectoderm	42.5±4.8	75.5±12.0	83.8±7.2
Percentage of ICM cells	33.4±3.0	34.9±3.2	17.8±2.7

Biology Open • Supplementary information

Table S3. Number and composition of samples from balanced series of E12.5 chimaeras.

Sample	All conceptuses	Chimaeric conceptuses			
	A : CHIM : B (number)	a : chim : b (number)	%GPI1A (incl NCS) <25 : 25-75: >75	<50%A : >50%A (% >50%A) incl NCS[‡]	excl NCS[‡]
**Combined Balanced Series XM, XP, PCT-VI and GMA**					
Fetus	6 : 115 : 9	9 : 103 : 3	27 : 62 : 26	61 : 54 (47.0)	58 : 45 (43.7)
Amnion	5 : 115 : 9	9 : 103 : 3	30 : 64 : 21	60 : 55 (47.8)	57 : 46 (44.7)
YSM	5 : 114 : 9	9 : 103 : 2	29 : 63 : 22	63 : 51 (44.7)	61 : 42 (40.8)
Epi mean	6 : 115 : 9	6 : 109 : 0	28 : 66 : 21	61 : 54 (47.0)	61 : 48 (44.0)
YSE	4 : 114 : 9	1 : 104 : 9	33 : 76 : 5	82 : 32 (28.1)****	73 : 31 (29.8)****
Placenta	6 : 115 : 9	12 : 88 : 15	48 : 30 : 37	68 : 47 (40.9)	53 : 35 (39.8)
**Balanced Series XM**					
Fetus	2 : 33 : 2	1 : 32 : 0	3 : 24 : 6	18 : 15 (45.5)	18 : 14 (43.8)
Amnion	2 : 33 : 2	1 : 32 : 0	6 : 24 : 3	18 : 15 (45.5)	18 : 14 (43.8)
YSM	1 : 32 : 2	1 : 31 : 0	5 : 25 : 2	18 : 14 (43.8)	18 : 13 (41.9)
Epi mean	2 : 33 : 2	1 : 32 : 0	4 : 28 : 1	19 : 14 (42.4)	19 : 13 (40.6)
YSE	1 : 32 : 2	0 : 29 : 3	8 : 22 : 2	20 : 12 (37.5)	17 : 12 (41.4)
Placenta	2 : 33 : 2	0 : 32 : 1	14 : 10 : 9	20 : 13 (39.4)	19 : 13 (40.6)
**Balanced Series XP**					
Fetus	1 : 32 : 4	3 : 28 : 1	11 : 12 : 9	18 : 14 (43.8)	17 : 11 (39.3)
Amnion	1 : 32 : 4	2 : 29 : 1	11 : 15 : 6	19 : 13 (40.6)	18 : 11 (37.9)
YSM	1 : 32 : 4	1 : 31 : 0	11 : 16 : 5	20 : 12 (37.5)	20 : 11 (35.5)
Epi mean	1 : 32 : 4	1 : 31 : 0	11 : 14 : 7	18 : 14 (43.8)	18 : 13 (41.9)
YSE	1 : 32 : 4	1 : 28 : 3	13 : 18 : 1	22 : 10 (31.3)[†]	19 : 9 (32.1)
Placenta	1 : 32 : 4	1 : 24 : 7	16 : 6 : 10	20 : 12 (37.5)	13 : 11 (45.8)
**Balanced Series PCT-VI**					
Fetus	1 : 30 : 1	2 : 27 : 1	8 : 15 : 7	15 : 15 (50.0)	14 : 13 (48.1)
Amnion	1 : 30 : 1	2 : 26 : 2	6 : 16 : 8	13 : 17 (56.7)	11 : 15 (57.7)
YSM	1 : 30 : 1	3 : 27 : 0	7 : 13 : 10	14 : 16 (53.3)	14 : 13 (48.1)
Epi mean	1 : 30 : 1	1 : 29 : 0	7 : 15 : 8	14 : 16 (53.3)	14 : 15 (51.7)
YSE	1 : 30 : 1	0 : 29 : 1	4 : 26 : 0	25 : 5 (16.7)***	24 : 5 (17.2)***
Placenta	1 : 30 : 1	5 : 22 : 3	13 : 10 : 7	21 : 9 (30.0)*	18 : 4 (18.2)**
**Balanced Series GMA**					
Fetus	2 : 20 : 2	3 : 16 : 1	5 : 11 : 4	10 : 10 (43.8)	9 : 7 (43.8)
Amnion	1 : 20 : 2	4 : 16 : 0	7 : 8 : 5	10 : 10 (37.5)	10 : 6 (37.5)
YSM	2 : 20 : 2	4 : 14 : 2	6 : 9 : 5	11 : 9 (35.7)	9 : 5 (35.7)
Epi mean	2 : 20 : 2	3 : 17 : 0	6 : 9 : 5	10 : 10 (41.2)	10 : 7 (41.2)
YSE	1 : 20 : 2	0 : 18 : 2	8 : 10 : 2	15 : 5 (27.8)*	13 : 5 (27.8)
Placenta	2 : 20 : 2	6 : 10 : 4	5 : 4 : 11	7 : 13 (70.0)	3 : 7 (70.0)

Abbreviations: A, GPI1A non-chimaeric conceptus; a, GPI1A non-chimaeric sample; B, GPI1B non-chimaeric conceptus; b, GPI1B non-chimaeric sample; CHIM, chimaeric conceptus (GPI1A + GPI1B); chim, chimaeric sample (GPI1A + GPI1B); Epi, epiblast; excl, excluding; incl, including; NCS, non-chimaeric samples from chimaeric conceptuses; YSE, yolk sac endoderm; YSM, yolk sac mesoderm.

Distributions were classified as "balanced" if the numbers of individuals with <50% GPI1A and >50% GPI1A did not differ significantly and "typical" if more individuals had 25–75% GPI1A than either <25% or >75%. For example, in the combined series XM, XP, PCT-VI & GMA, fetuses, amnions, YSM samples and the epiblast means were all balanced and typical whereas YSE samples were unbalanced but typical and placentas were balanced but atypical.

[‡] The frequencies of samples from chimaeric conceptuses with <50% versus >50% GPI1A were compared to the 1:1 frequency expectations for a balanced distribution by a chi square goodness of fit test and corrected for continuity. * $P<0.05$; ** $P<0.01$; *** $P<0.001$; **** $P<0.0001$. ([†]$P \geq 0.05$ if chi square is corrected for continuity but $P < 0.05$ for uncorrected chi square.)

Table S4. Number and composition of samples from unbalanced series of E12.5 chimaeras.

Sample	All conceptuses	Chimaeric conceptuses			
	A : CHIM : B (number)	a : chim : b (number)	%GPI1A (incl NCS) <25 : 25-75: >75	<50%A : >50%A (% >50%A) incl NCS[‡]	<50%A : >50%A (% >50%A) excl NCS[‡]
**Combined Unbalanced Series XR, XN, PCT-V and GMB**					
Fetus	5 : 118 : 32	2 : 78 : 38	70 : 41 : 7	96 : 22 (18.6)****	58 : 20 (25.6)****
Amnion	5 : 117 : 31	2 : 76 : 39	76 : 35 : 6	100 : 17 (14.5)****	61 : 15 (19.7)****
YSM	4 : 117 : 32	1 : 78 : 38	73 : 40 : 4	99 : 18 (15.4)****	61 : 17 (21.8)****
Epi mean	5 : 118 : 32	1 : 81 : 36	72 : 41 : 5	99 : 19 (16.1)****	63 : 18 (22.2)****
YSE	4 : 118 : 32	0 : 102 : 16	65 : 50 : 3	105 : 13 (11.0)****	89 : 13 (12.7)****
Placenta	5 : 118 : 32	6 : 60 : 52	76 : 25 : 17	91 : 27 (22.9)****	39 : 21 (35.0)*
**Unbalanced Series XR**					
Fetus	2 : 38 : 12	0 : 18 : 20	29 : 7 : 2	34 : 4 (10.5)****	14 : 4 (22.2)*
Amnion	2 : 38 : 12	0 : 17 : 21	31 : 5 : 2	34 : 4 (10.5)****	13 : 4 (23.5)[†]
YSM	2 : 38 : 12	0 : 17 : 21	30 : 7 : 1	34 : 4 (10.5)****	13 : 4 (23.5)[†]
Epi mean	2 : 38 : 12	0 : 19 : 19	30 : 6 : 2	34 : 4 (10.5)****	15 : 4 (21.1)*
YSE	2 : 38 : 12	0 : 31 : 7	28 : 10 : 0	37 : 1 (2.6)****	30 : 1 (3.2)****
Placenta	2 : 38 : 12	2: 12 : 24	30 : 5 : 3	31 : 7 (18.4)***	7 : 5 (41.7)
**Unbalanced Series XN[§]**					
Fetus	2 : 29 : 9	0 : 25 : 4	14 : 13 : 2	20 : 9 (31.0)[†]	16 : 9 (36.0)
Amnion	2 : 29 : 9	0 : 25 : 4	12 : 15 : 2	22 : 7 (24.1)**	18 : 7 (28.0)*
YSM	2 : 29 : 9	0 : 26 : 3	14 : 14 : 1	23 : 6 (20.7)**	20 : 6 (23.1)*
Epi mean	2 : 29 : 9	0 : 26 : 3	12 : 16 : 1	22 : 7 (24.1)**	19 : 7 (26.9)*
YSE	2 : 29 : 9	0 : 25 : 4	14 : 13 : 2	22 : 7 (24.1)**	18 : 7 (28.0)*
Placenta	2 : 29 : 9	1 : 21 : 7	17 : 7 : 5	22 : 7 (24.1)**	15 : 6 (28.6)[†]
**Unbalanced Series PCT-V**					
Fetus	0 : 31 : 4	0 : 21 : 10	19 : 11 : 1	26 : 5 (16.1)***	16 : 5 (23.8)*
Amnion	0 : 31 : 4	0 : 21 : 10	22 : 9 : 0	28 : 3 (9.7)****	18 : 3 (14.3)**
YSM	0 : 31 : 4	0 : 21 : 10	21 : 10 : 0	26 : 5 (16.1)***	16 : 5 (23.8)*
Epi mean	0 : 31 : 4	0 : 21 : 10	21 : 10 : 0	26 : 5 (16.1)***	16 : 5 (23.8)*
YSE	0 : 31 : 4	0 : 27 : 4	11 : 19 : 1	28 : 3 (9.7)****	24 : 3 (11.1)***
Placenta	0 : 31 : 4	0 : 18 : 13	21 : 6 : 4	25 : 6 (19.4)**	12 : 6 (33.3)
**Unbalanced Series GMB**					
Fetus	1 : 20 : 7	2 : 14 : 4	8 : 10 : 2	16 : 4 (20.0)*	12 : 2 (14.3)*
Amnion	1 : 19 : 6	2 : 13 : 4	11 : 6 : 2	16 : 3 (15.8)**	12 : 1 (7.7)**
YSM	0 : 19 : 7	1 : 14 : 4	8 : 9 : 2	16 : 3 (15.8)**	12 : 2 (14.3)*
Epi mean	1 : 20 : 7	1 : 15 : 4	9 : 9 : 2	17 : 3 (15.0)**	13 : 2 (13.3)
YSM	0 : 20 : 7	0 : 19 : 1	12 : 8 : 0	18 : 2 (10.0)***	17 : 2 (10.5)**
Placenta	1 : 20 : 7	3 : 9 : 8	8 : 7 : 5	13 : 7 (35.0)**	5 : 4 (44.4)

Abbreviations: A, GPI1A non-chimaeric conceptus; a, GPI1A non-chimaeric sample; B, GPI1B non-chimaeric conceptus; b, GPI1B non-chimaeric sample; CHIM, chimaeric conceptus (GPI1A + GPI1B); chim, chimaeric sample (GPI1A + GPI1B); Epi, epiblast; excl, excluding; incl, including; NCS, non-chimaeric samples from chimaeric conceptuses; YSE, yolk sac endoderm; YSM, yolk sac mesoderm.

Distributions were classified as “balanced” if the numbers of individuals with <50% GPI1A and >50% GPI1A did not differ significantly and “typical” if more individuals had 25–75% GPI1A than either <25% or >75%.

[‡]The frequencies of samples from chimaeric conceptuses with <50% versus >50% GPI1A were compared to the 1:1 frequency expectations for a balanced distribution by a chi square goodness of fit test and corrected for continuity. * $P<0.05$; ** $P<0.01$; *** $P<0.001$; **** $P<0.0001$. ([†]$P \geq 0.05$ if chi square is corrected for continuity but $P < 0.05$ for uncorrected chi square.)

[§] Series XN was considered to be unbalanced because, although the imbalance in series XN fetal samples was mild, the XN Epi mean, amnion and YSM distributions were all unbalanced according to the criterion that the numbers of individuals with <50% GPI1A differed significantly from those with >50% GPI1A.

Table S5. Comparison of frequencies of non-chimaeric conceptuses between pooled balanced and pooled unbalanced series of E12.5 conceptuses.

Sample	NCC : CC (% Non-chimaeric)		NCC-A : NCC-B (% Non-chimaeric GPI1A)	
	**Balanced**	**Unbalanced**	**Balanced**	**Unbalanced**
**Non-chimaeric and Chimaeric Conceptuses**				
Conceptus	15 : 115 (11.5)	37 : 118 (23.9)**	6 : 9 (40.0)	5 : 32 (13.5)

Abbreviations: CC, chimaeric conceptus; NCC, non-chimaeric conceptus; NCC-A, non-chimaeric conceptus with only GPI1A; NCC-B, non-chimaeric conceptus with only GPI1B.

The NCC : CC and NCC-A : NCC-B frequencies were compared between balanced and unbalanced series of chimaeras by Fisher's exact test. ** $P < 0.01$.

Table S6. Comparison of frequencies of non-chimaeric samples between pooled balanced and pooled unbalanced series of E12.5 chimaeras.

Sample	NCS : CS (% Non-chimaeric)		NCS-A : NCS-B (% Non-chimaeric GPI1A)	
	**Balanced**	**Unbalanced**	**Balanced**	**Unbalanced**
**Non-chimaeric and Chimaeric Samples (from chimaeric conceptuses)**				
Fetus	12 : 103 (10.4)	40 : 78 (33.9)****	9 : 3 (75.0)	2 : 38 (5.0)****
Amnion	12 : 103 (10.4)	41 : 76 (35.0)****	9 : 3 (75.0)	2 : 39 (4.9)****
YS mesoderm	11 : 103 (9.6)	39 : 78 (33.3)****	9 : 2 (81.8)	1 : 38 (2.6)****
Epi mean	6 : 109 (5.2)	37 : 81 (31.4)****	6 : 0 (100.0)	1 : 36 (2.7)****
YS endoderm	10 : 104 (8.8)	16 : 102 (13.6)	1 : 9 (10.0)	0 : 16 (0.0)
Placenta	27 : 88 (23.5)	58 : 60 (49.2)****	12 : 15 (44.4)	6 : 52 (10.3)**

Abbreviations: CS, chimaeric sample; NCS, non-chimaeric sample; NCS-A, non-chimaeric sample with only GPI1A; NCS-B, non-chimaeric sample with only GPI1B; YS, Yolk Sac.

The NCS : CS and NCS-A : NCS-B frequencies were compared between balanced and unbalanced series of chimaeras by Fisher's exact test. * $P < 0.05$; ** $P < 0.01$; *** $P < 0.001$; **** $P < 0.0001$.

Table S7. Spearman correlation coefficient matrix for the percentage GPI1A in samples from four balanced series of E12.5 chimaeras.

A. Correlations for individual series of balanced chimaeras

Spearman correlation coefficients (r_s) for balanced series XM (N = 33)

	Fetus	Amnion	YSM	Epi mean	YSE	Par end
**Amnion**	0.880****					
**YSM**	0.934****	0.885****				
**Epi mean**	0.974****	0.942****	0.967****			
**YSE**	0.190	0.220	0.137	0.168		
**Par end**	0.089	0.039	0.038	0.054	0.517**	
**Placenta**	0.345*	0.258	0.324	0.348*	0.353*	-0.061

Spearman correlation coefficients (r_s) for balanced series XP (N = 32)

	Fetus	Amnion	YSM	Epi mean	YSE	Par end
**Amnion**	0.981****					
**YSM**	0.957****	0.959****				
**Epi mean**	0.989****	0.989****	0.977****			
**YSE**	0.263	0.261	0.393*	0.288		
**Par end**	0.426*	0.406*	0.453**	0.408*	0.674****	
**Placenta**	0.650****	0.664****	0.559***	0.626***	0.131	0.195

Spearman correlation coefficients (r_s) for balanced series PCT-VI (N = 30)

	Fetus	Amnion	YSM	Epi mean	YSE	Par end
**Amnion**	0.941****					
**YSM**	0.961****	0.929****				
**Epi mean**	0.984****	0.973****	0.977****			
**YSE**	0.442*	0.476**	0.407*	0.435*		
**Par end**	ND	ND	ND	ND	ND	
**Placenta**	0.439*	0.459*	0.424*	0.424*	0.558**	ND

Spearman correlation coefficients (r_s) for balanced series GMA (N = 20)

	Fetus	Amnion	YSM	Epi mean	YSE	Par end
**Amnion**	0.871****					
**YSM**	0.907****	0.906****				
**Epi mean**	0.938****	0.949****	0.975****			
**YSE**	0.653**	0.574**	0.664**	0.644**		
**Par end**	ND	ND	ND	ND	ND	
**Placenta**	0.740***	0.730***	0.703***	0.755***	0.684***	ND

B. Correlations from pooled combinations of balanced chimaeras

Spearman correlation coefficients (r_s) for balanced series XM & XP (N = 65)

	Fetus	Amnion	YSM	Epi mean	YSE	Par end
**Amnion**	0.944****					
**YSM**	0.953****	0.941****				
**Epi mean**	0.984****	0.978****	0.978****			
**YSE**	0.248*	0.247*	0.285*	0.259*		
**Par end**	0.266*	0.219	0.271**	0.247*	0.617****	
**Placenta**	0.536****	0.518****	0.489****	0.528****	0.218	0.069

Spearman correlation coefficients (r_s) for balanced series XM, XP, PCT-VI & GMA (N = 115)

	Fetus	Amnion	YSM	Epi mean	YSE	Par end
**Amnion**	0.916****					
**YSM**	0.944****	0.936****				
**Epi mean**	0.973****	0.974****	0.980****			
**YSE**	0.368****	0.380****	0.411****	0.394****		
**Par end**	ND	ND	ND	ND	ND	
**Placenta**	0.543****	0.501****	0.500****	0.529****	0.360****	ND

* $P < 0.05$; ** $P < 0.01$; *** $P < 0.001$; **** $P < 0.0001$. Negative Spearman correlation coefficients are shown in red. Abbreviations: Epi, epiblast; ND, not done; Par end, parietal endoderm; YSE, yolk sac endoderm; YSM, yolk sac mesoderm.

Table S8. Spearman correlation coefficient matrix for the percentage GPI1A in samples from four unbalanced series of E12.5 chimaeras.

A. Correlations for individual series of balanced chimaeras

Spearman correlation coefficients (r_s) for unbalanced series XR (N = 38)

	Fetus	Amnion	YSM	Epi mean	YSE	Par end
**Amnion**	0.914****					
**YSM**	0.970****	0.948****				
**Epi mean**	0.979****	0.963****	0.970****			
**YSE**	0.089	0.110	0.120	0.070		
**Par end**	0.263	0.244	0.289	0.261	0.557***	
**Placenta**	0.625****	0.596****	0.640****	0.627****	-0.049	0.196

Spearman correlation coefficients (r_s) for unbalanced series XN (N = 29)

	Fetus	Amnion	YSM	Epi mean	YSE	Par end
**Amnion**	0.945****					
**YSM**	0.943****	0.962****				
**Epi mean**	0.983****	0.977****	0.977****			
**YSE**	0.632***	0.628***	0.547**	0.620***		
**Par end**	0.615***	0.561**	0.534**	0.585**	0.777****	
**Placenta**	0.396*	0.490**	0.536**	0.481**	0.155	0.180

Spearman correlation coefficients (r_s) for unbalanced series PCT-V (N = 31)

	Fetus	Amnion	YSM	Epi mean	YSE	Par end
**Amnion**	0.975****					
**YSM**	0.982****	0.982****				
**Epi mean**	0.992****	0.988****	0.993****			
**YSE**	0.377*	0.357*	0.382*	0.371*		
**Par end**	ND	ND	ND	ND	ND	
**Placenta**	0.409*	0.452*	0.442*	0.449*	-0.089	ND

Spearman correlation coefficients (r_s) for unbalanced series GMB (N = 20)

	Fetus	Amnion	YSM	Epi mean	YSE	Par end
**Amnion**	0.853****					
**YSM**	0.861****	0.926****				
**Epi mean**	0.916****	0.980****	0.959****			
**YSE**	0.283	0.369	0.525*	0.392		
**Par end**	ND	ND	ND	ND	ND	
**Placenta**	0.556*	0.640**	0.541*	0.621**	0.324	ND

B. Correlations from pooled combinations of balanced chimaeras

Spearman correlation coefficients (r_s) for unbalanced series XR & XN (N=67)

	Fetus	Amnion	YSM	Epi mean	YSE	Par end
**Amnion**	0.956****					
**YSM**	0.964****	0.963****				
**Epi mean**	0.985****	0.980****	0.984****			
**YSE**	0.448***	0.455***	0.419***	0.424***		
**Par end**	0.423***	0.384**	0.391**	0.398***	0.661****	
**Placenta**	0.612****	0.639****	0.681****	0.661****	0.1771	0.251*

Spearman correlation coefficients (r_s) for unbalanced series XR, XN, PCT-V & GMB (N=118)

	Fetus	Amnion	YSM	Epi mean	YSE	Par end
**Amnion**	0.954****					
**YSM**	0.962****	0.970****				
**Epi mean**	0.984****	0.985****	0.988****			
**YSE**	0.434****	0.434****	0.435****	0.425****		
**Par end**	ND	ND	ND	ND	ND	
**Placenta**	0.550****	0.601****	0.610****	0.601****	0.149	ND

* *P* < 0.05; ** *P* < 0.01; *** *P* < 0.001; **** *P* < 0.0001. Negative Spearman correlation coefficients are shown in red. Abbreviations: Epi, epiblast; ND, not done; Par end, parietal endoderm; YSE, yolk sac endoderm; YSM, yolk sac mesoderm.

Biology Open • Supplementary information

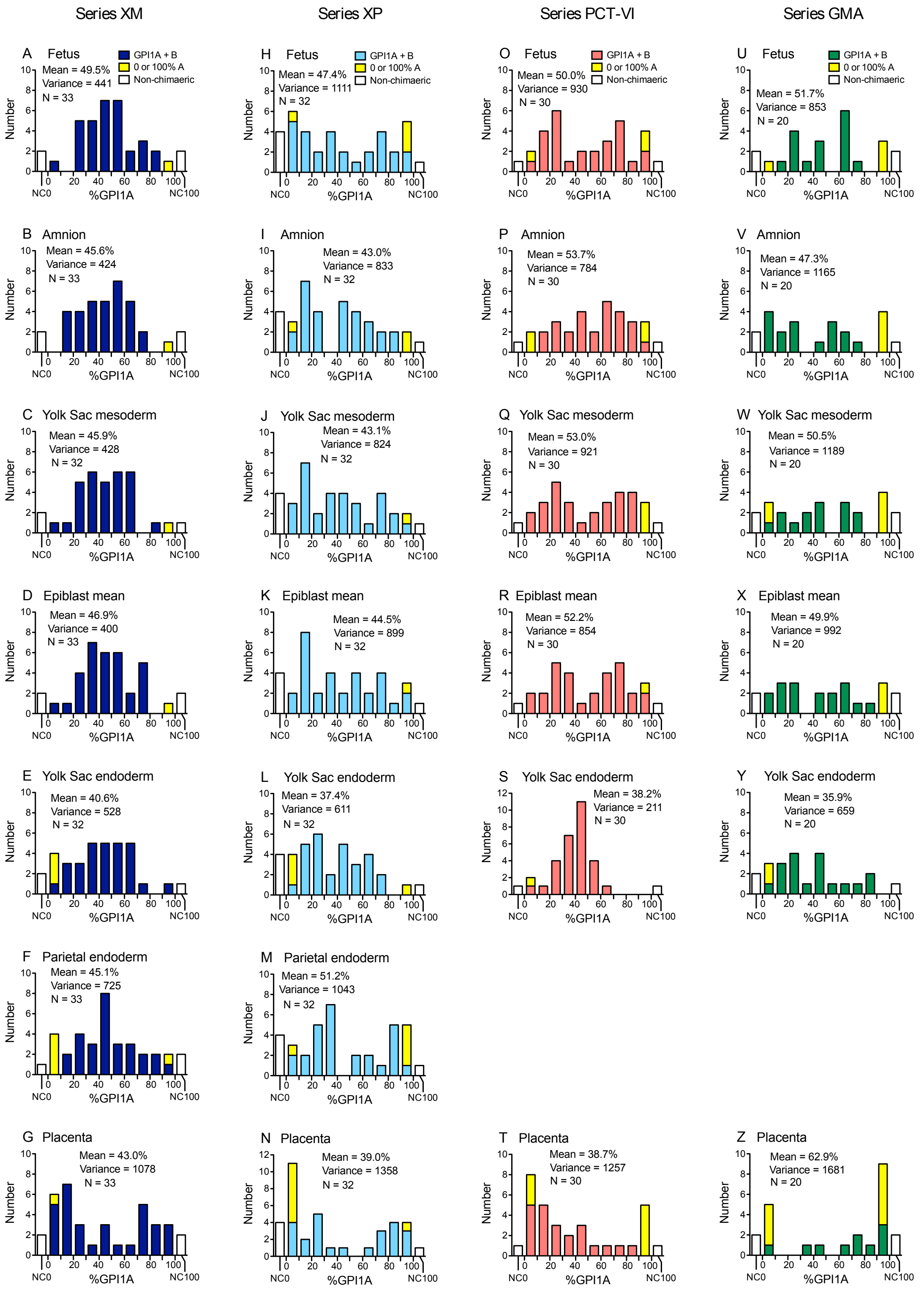

Fig. S1. Frequency distributions of the percentage GPI1A in different samples from four balanced series of E12.5 chimaeric conceptuses.

Percentage GPI1A frequency distributions for four published series of aggregation chimaeras: **(A–G)** series XM, **(H–N)** XP, **(O–T)** PCT-VI and **(U–Z)** GMA. Non-chimaeric conceptuses had 0% or 100% GPI1A in the fetus and all extraembryonic samples and are shown as white bars at the ends of the distributions. Non-chimaeric samples from chimaeric conceptuses are shown as yellow bars with the 0-10% GPI1A or 90-100% GPI1A group as appropriate. The mean, variance and number of samples (N), shown in the figures, exclude non-chimaeric conceptuses. See Table S1 for details of chimaera strain combinations and references to original publications. Parietal endoderm samples were not analysed for series PCT-VI or GMA. Abbreviations: NC0, non-chimaeric with 0% GPI1A; NC100, non-chimaeric with 100% GPI1A.

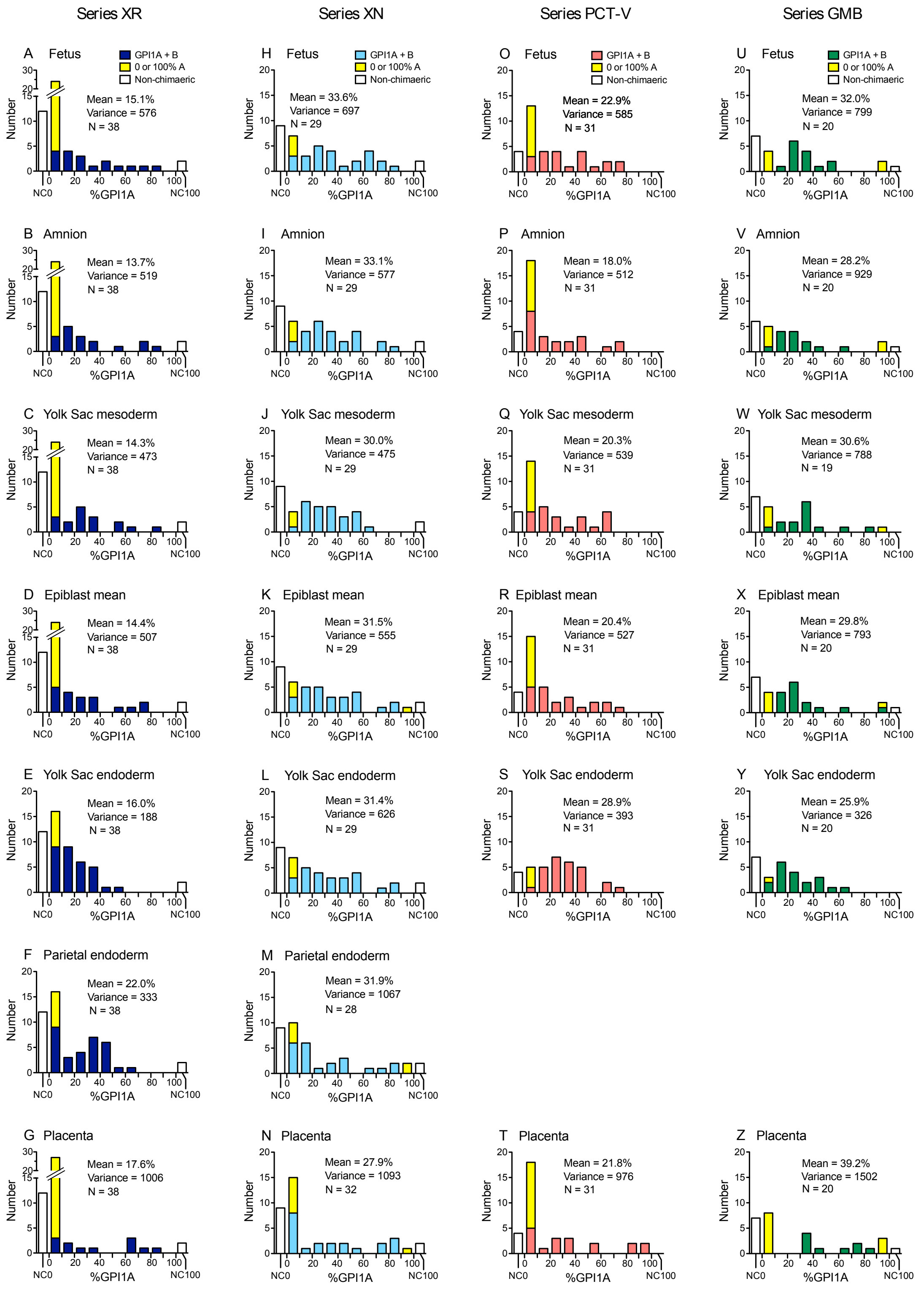

Fig. S2. Frequency distributions of the percentage GPI1A in different samples from four unbalanced series of E12.5 chimaeric conceptuses.

Percentage GPI1A frequency distributions for four published series of aggregation chimaeras: **(A–G)** series XR, **(H–N)** XN, **(O–T)** PCT-V and **(U–Z)** GMB. Non-chimaeric conceptuses had 0% or 100% GPI1A in the fetus and all extraembryonic samples and are shown as white bars at the ends of the distributions. Non-chimaeric samples from chimaeric conceptuses are shown as yellow bars with the 0–10% GPI1A or 90–100% GPI1A group as appropriate. The mean, variance and number of samples (N), shown in the figures, exclude non-chimaeric conceptuses. See Table S1 for details of chimaera strain combinations and references to original publications. Parietal endoderm samples were not analysed for series PCT-V or GMB. Abbreviations: NC0, non-chimaeric with 0% GPI1A; NC100, non-chimaeric with 100% GPI1A.

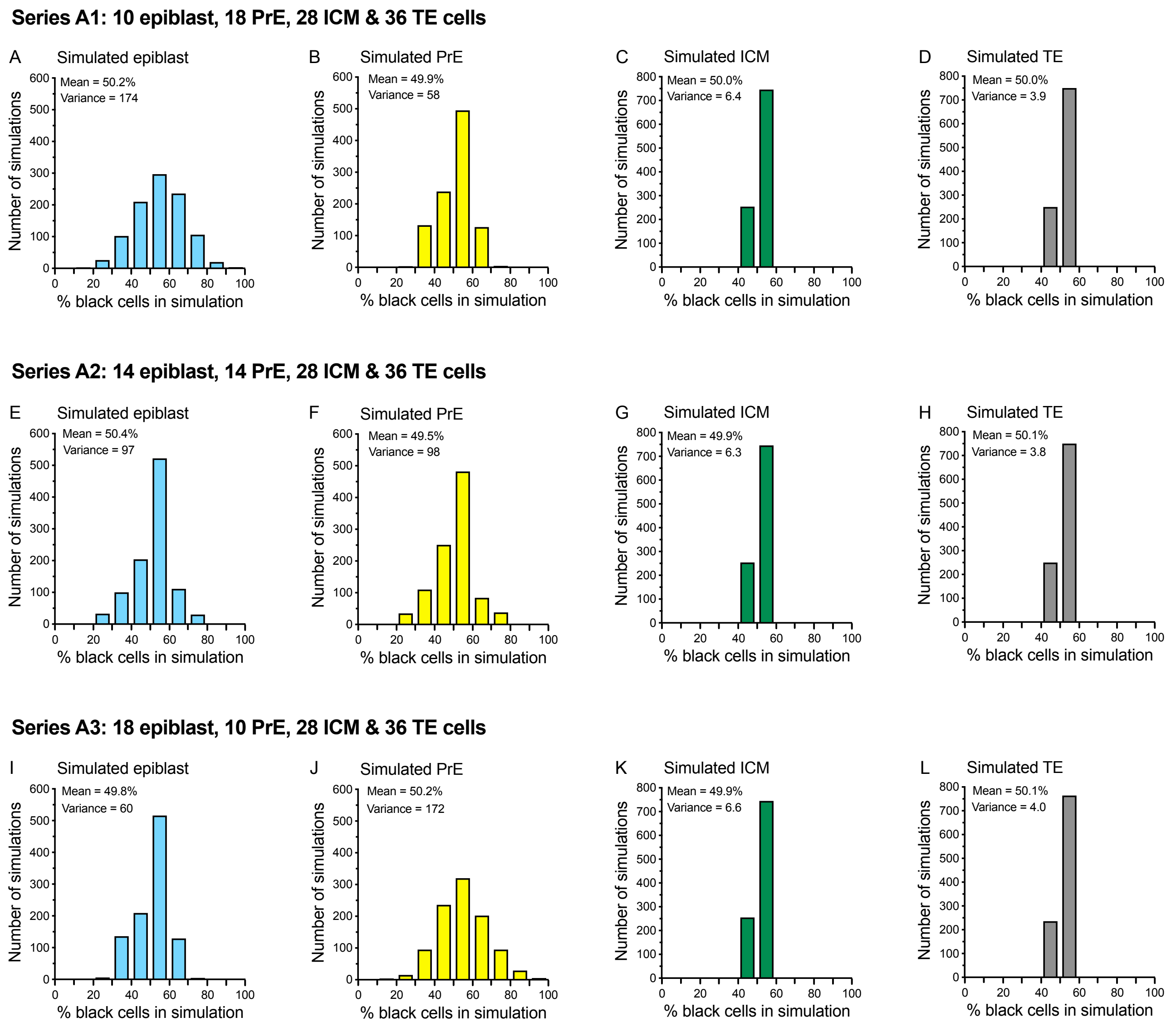

Fig. S3. Variation in percentage of black cells in simulated chimaeric blastocyst lineages from simulation model A.

Frequency distributions for the percentage of black cells in simulated lineages (epiblast, PrE, ICM and TE) from a representative set of 1000 simulated 64-cell, chimaeric blastocysts for each of three simulation series (A1–A3) for model A. (The set of simulations chosen to be representative had an epiblast variance that was closest to the mean epiblast variance for the 10 sets in that series.) In this model, little variation was introduced at allocation step 1 (all ICMs and TEs had 46.4–53.6% black cells) but more variation was introduced at allocation step 2 when ICM cells were allocated to the epiblast and PrE. No cell death was simulated and all simulated blastocysts had 50% black and 50% white cells overall. All simulated blastocysts had 28 ICM cells and 36 TE cells but the numbers of epiblast and PrE cells varied among series A1–A3. (**A–D**) Series A1 with 10 epiblast cells and 18 PrE cells; (**E–H**) Series A2 with 14 epiblast cells and 14 PrE cells; (**I–L**) Series A3 with 18 epiblast cells and 10 PrE cells. The mean percentage of black cells and its variance are shown in the figure.

Biology Open • Supplementary information

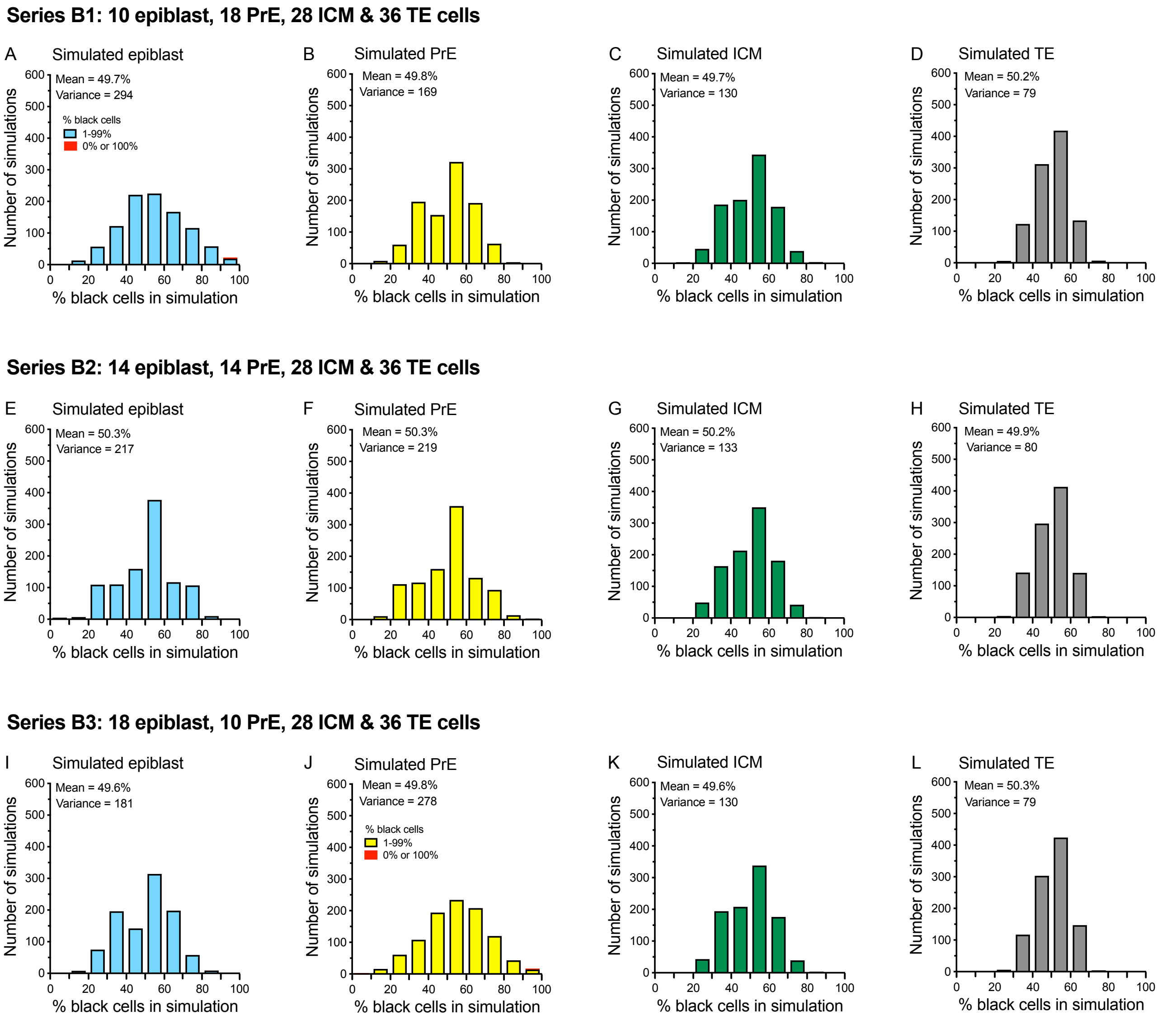

Fig. S4. Variation in percentage of black cells in simulated chimaeric blastocyst lineages from simulation model B.

Frequency distributions for the percentage of black cells in simulated lineages (epiblast, PrE, ICM and TE) from a representative set of 1000 simulated 64-cell, chimaeric blastocysts for each of three simulation series (B1–B3) for 2-step model B. (The set of simulations chosen to be representative had an epiblast variance that was closest to the mean epiblast variance for the 10 sets in that series.) In this model, significant variation was introduced at both allocation step 1 (when cells were allocated to the TE and ICM) and allocation step 2 (when ICM cells were allocated to the epiblast and PrE). No cell death was simulated and all simulated blastocysts had 50% black and 50% white cells overall. All simulated blastocysts had 28 ICM cells and 36 TE cells but the numbers of epiblast and PrE cells varied among series B1–B3. (**A–D**) Series B1 with 10 epiblast cells and 18 PrE cells; (**E–H**) Series B2 with 14 epiblast cells and 14 PrE cells; (**I–L**) Series B3 with 18 epiblast cells and 10 PrE cells. The mean percentage of black cells and its variance are shown in the figure.

Biology Open • Supplementary information

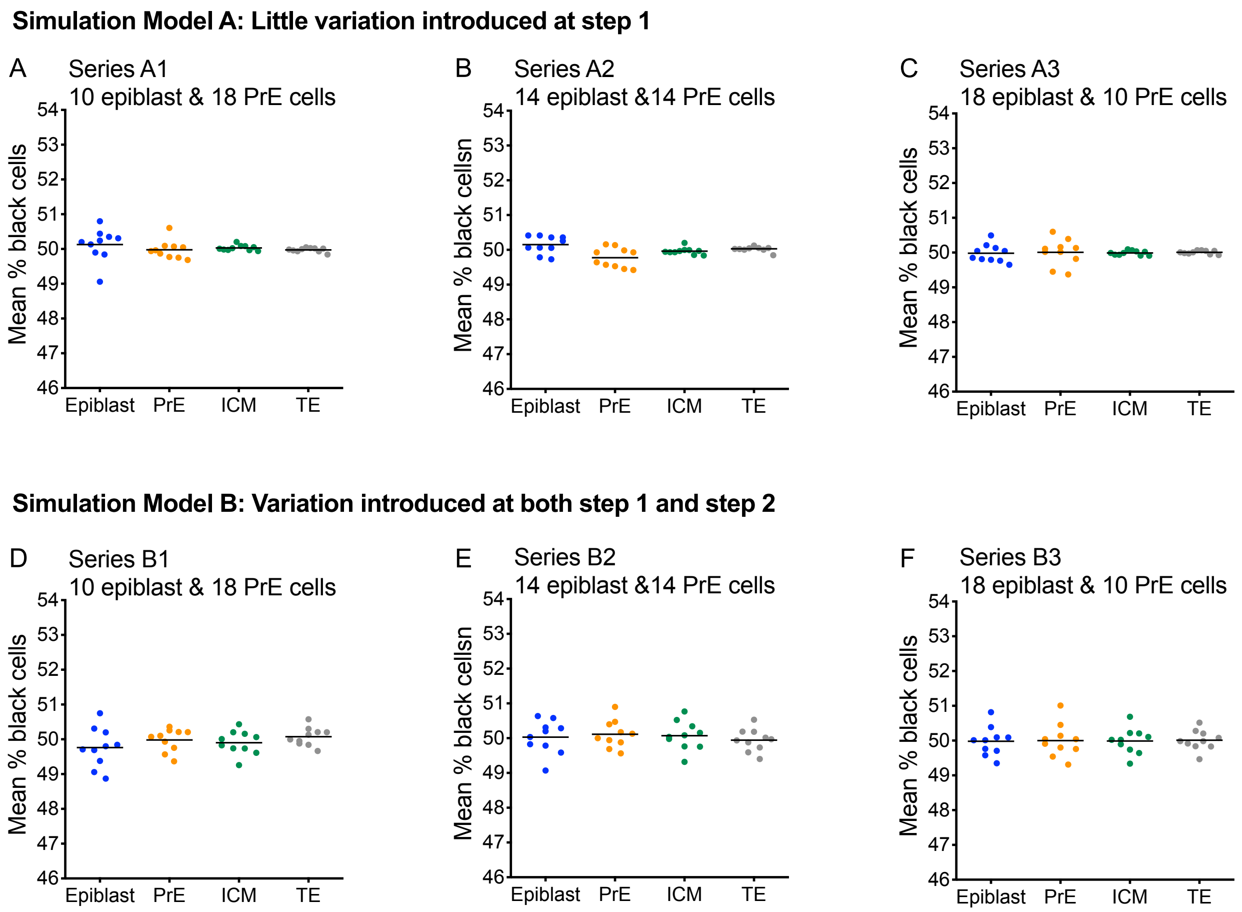

Fig. S5. Comparison of percentage of black cells in simulated chimaeric blastocyst lineages in six series of simulations.

Scatter plots for the mean percentage of black cells in simulated lineages for ten sets of 1000 simulated 64-cell, chimaeric blastocysts for each of six simulation series, A1–A3 and B1–B3. (Each point on the graph represents the mean percentage of black cells for a set of 1000 simulated chimaeric blastocysts.) No cell death was simulated and all simulated blastocysts had 50% black and 50% white cells overall, 28 ICM cells and 36 TE cells. However, the numbers of epiblast and PrE cells varied among series A1–A3 and among series B1–B3. (**A–C**) Results for series A1–A3 from simulation model A, in which little variation was introduced at allocation step 1, when cells were allocated to the TE and ICM (all ICMs and TEs had 46.4–53.6% black cells) but more variation was introduced at allocation step 2, when ICM cells were allocated to the epiblast and PrE. (**A**) Series A1 with 10 epiblast cells and 18 PrE cells; (**B**) Series A2 with 14 epiblast cells and 14 PrE cells; (**C**) Series A3 with 18 epiblast cells and 10 PrE cells. (**D–F**) Results for series B1–B3 from simulation model B, in which significant variation was introduced at both allocation steps 1 and 2. (**D**) Series B1 with 10 epiblast cells and 18 PrE cells; (**E**) Series B2 with 14 epiblast cells and 14 PrE cells; (**F**) Series B3 with 18 epiblast cells and 10 PrE cells.

Biology Open • Supplementary information

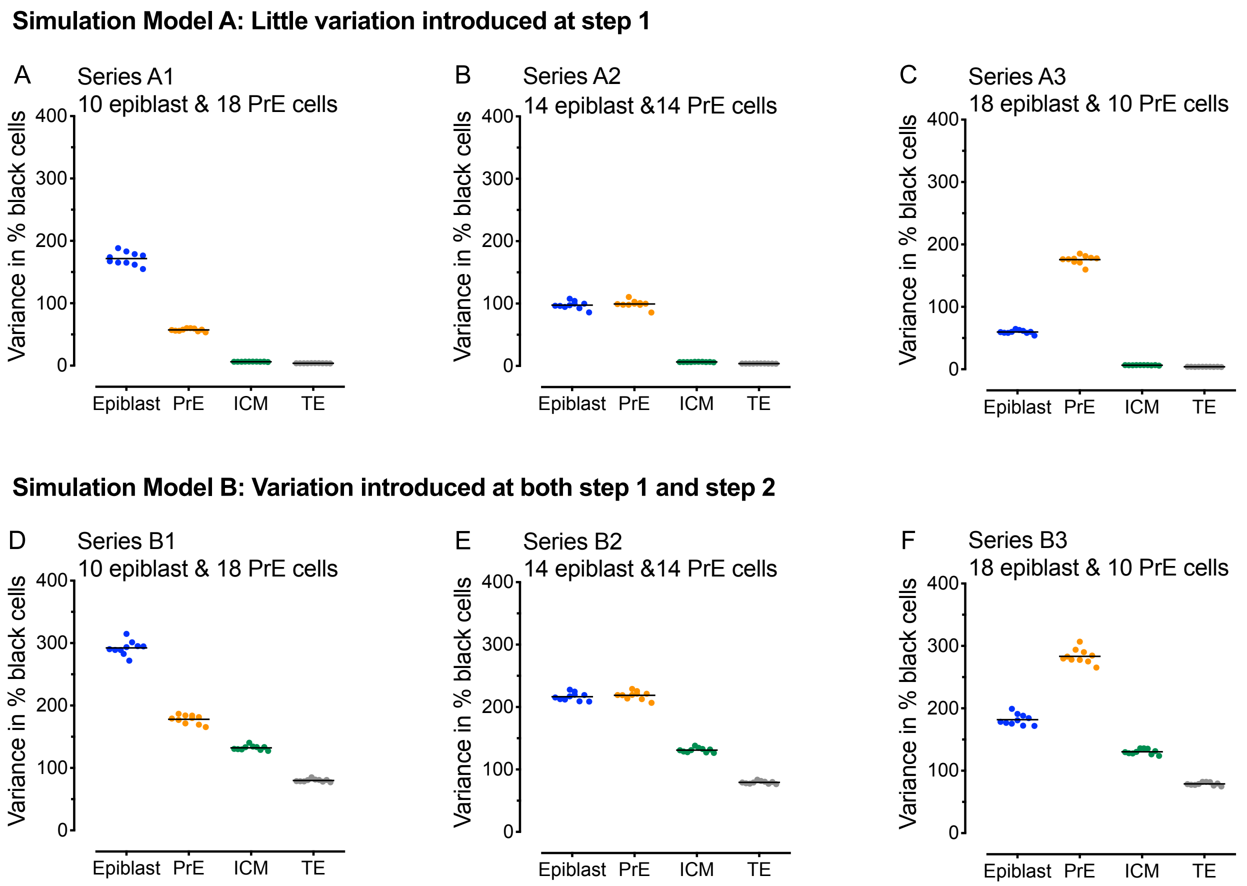

Fig. S6. Comparison of variance in percentage of black cells in simulated chimaeric blastocyst lineages in six series of simulations.

Scatter plots for the variance in percentage of black cells in simulated lineages for ten sets of 1000 simulated 64-cell, chimaeric blastocysts for each of six simulation series, A1–A3 and B1–B3. (Each point on the graph represents the variance for a set of 1000 simulated chimaeric blastocysts.) No cell death was simulated and all simulated blastocysts had 50% black and 50% white cells overall, 28 ICM cells and 36 TE cells. However, the numbers of epiblast and PrE cells varied among series A1–A3 and among series B1–B3. (**A–C**) Results for series A1–A3 from simulation model A, in which little variation was introduced at allocation step 1, when cells were allocated to the TE and ICM (all ICMs and TEs had 46.4–53.6% black cells) but more variation was introduced at allocation step 2, when ICM cells were allocated to the epiblast and PrE. (**A**) Series A1 with 10 epiblast cells and 18 PrE cells; (**B**) Series A2 with 14 epiblast cells and 14 PrE cells; (**C**) Series A3 with 18 epiblast cells and 10 PrE cells. (**D–F**) Results for series B1–B3 from simulation model B, in which significant variation was introduced at both allocation steps 1 and 2. (**D**) Series B1 with 10 epiblast cells and 18 PrE cells; (**E**) Series B2 with 14 epiblast cells and 14 PrE cells; (**F**) Series B3 with 18 epiblast cells and 10 PrE cells.

Biology Open • Supplementary information
